# Aperiodic and Hurst EEG exponents across early human brain development: A systematic review

**DOI:** 10.1016/j.dcn.2024.101402

**Published:** 2024-06-06

**Authors:** R.A. Stanyard, D. Mason, C. Ellis, H. Dickson, R. Short, D. Batalle, T. Arichi

**Affiliations:** a Centre for the Developing Brain, School of Biomedical Engineering and Imaging Sciences, King's College London, United Kingdom; b Department of Forensic and Neurodevelopmental Sciences, Institute of Psychiatry, Psychology and Neuroscience, King’s College London, United Kingdom; c Social, Genetic, and Developmental Psychiatry Centre, Institute of Psychiatry, Psychology and Neuroscience, King’s College London, United Kingdom; d MRC Centre for Neurodevelopmental Disorders, King's College London, United Kingdom; e Children's Neurosciences, Evelina London Children's Hospital, Guy's and St Thomas' NHS Foundation Trust, United Kingdom; f Department of Bioengineering, Imperial College London, United Kingdom

**Keywords:** Systematic review, Aperiodic exponent, Hurst exponent, EEG, Electroencephalography, Development, Infant, Toddler, Child, Adolescent, Young adult

## Abstract

In electroencephalographic (EEG) data, power-frequency slope exponents (1/*f*^_β^) can provide non-invasive markers of *in vivo* neural activity excitation-inhibition (E:I) balance. E:I balance may be altered in neurodevelopmental conditions; hence, understanding how 1/*f*^β^ evolves across infancy/childhood has implications for developing early assessments/interventions. This systematic review (PROSPERO-ID: CRD42023363294) explored the early maturation (0–26 yrs) of resting-state EEG 1/*f* measures (aperiodic [AE], power law [PLE] and Hurst [HE] exponents), including studies containing ≥1 1/*f* measures and ≥10 typically developing participants. Five databases (including Embase and Scopus) were searched during March 2023. Forty-two studies were identified (N_participants_=3478). Risk of bias was assessed using the Quality Assessment with Diverse Studies tool. Narrative synthesis of HE data suggests non-stationary EEG activity occurs throughout development. Age-related trends were complex, with rapid decreases in AEs during infancy and heterogenous changes thereafter. Regionally, AE maxima shifted developmentally, potentially reflecting spatial trends in maturing brain connectivity. This work highlights the importance of further characterising the development of 1/*f* measures to better understand how E:I balance shapes brain and cognitive development.

## Highlights

• Complex changes occur to the magnitude of EEG aperiodic exponents in childhood.
• Aperiodic exponent topographic maxima shift from back to front from infancy to young adulthood.
• More infant and toddler 1/f studies are needed to understand ongoing 1/f trends.

## Graphical Abstract

**Figure:**
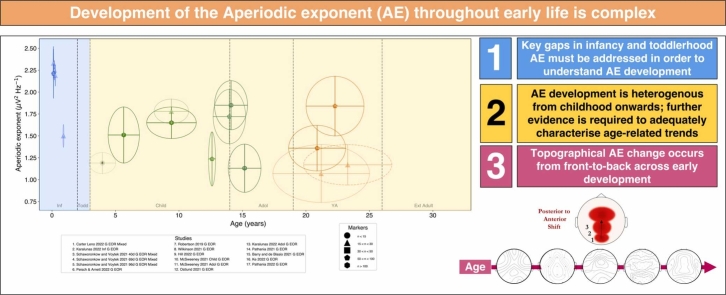

## 1 Introduction

The maintenance of excitation and inhibition (E:I) balance in the brain is an essential homeostatic mechanism that regulates spontaneous neural activity and facilitates the complex activity patterns thought to underlie efficient information processing and adaptive behaviour (Rocha, 2018, Bassi, 2019). It has been suggested that this key feature of brain physiology can be represented by a power law (1/*f*) relationship between spectral frequencies and spectral power in electrophysiological data (Boustani, 2009, Gao et al., 2017, Donoghue, 2020). Steeper 1/*f* profiles (higher exponents) characterised within specific frequency ranges (Manning, 2009, Miller, 2012) suggest higher contributions of inhibitory (*i.e.* increased GABAergic/decreased glutamatergic) signalling whereas flatter (lower) exponents suggest excitation-dominant signalling (E>I) (Gyurkovics et al., 2022). This can be non-invasively studied using electroencephalography [EEG] (Waschke et al., 2017) which is sensitive to local field potential (LFP) aggregates including faster decaying excitatory AMPA and slower decaying inhibitory GABA currents (Buzsáki et al., 2012); and thus changes in power spectral densities (PSDs) will affect estimated 1/*f* exponents. However, the biological link between 1/*f* and E:I is still under investigation (Gao et al., 2017, Salvatore, 2024).

The power spectrum can be further decomposed into both frequency-specific ‘periodic’ oscillations and an ‘aperiodic’ signal (termed *β* or χ) (Voytek and Kramer, 2015). In adulthood, *β* significantly declines with age (Voytek and Kramer, 2015, Waschke et al., 2017) although the physiological origin of this age-related change is unclear. Flattening of the 1/*f* slope has been associated with a reduction in the autocorrelation of brain activity, allowing for more efficient information processing (He, 2014). EEG 1/*f* measures also display behavioural and clinical relevance, particularly in conditions thought to relate to shifts in E:I balance, including those affecting selective attention and inhibition such as attention-deficit hyperactivity disorder (ADHD) (Waschke, 2021, Robertson, 2019). In addition, EEG 1/*f* have also been associated with states of consciousness (Leroy, 2023), and functional recovery from stroke (Leemburg, 2018). Prior to future research utilising 1/*f* measures to explore possible atypical brain E:I or exploring its potential use as a clinical “biomarker”, 1/*f* measures must first be characterised across the typically developing (TD) lifespan, from infancy to early adulthood (other studies have begun to chart this for later adulthood, see Finley, 2022).

Three different methods for deriving the 1/*f* ^β^ exist in the human EEG literature: (1) power law exponents (PLEs) (He, 2014) estimated from the slope of log-frequency versus log-power distributions and measures accounting for periodic oscillations, including aperiodic exponents (μV^2^ Hz^−1^) [AEs] which can be estimated using one of the following approaches; (2) fitting of one-over-f [FOOOF] (now specparams) via estimation of an initial slope and iterative estimation of gaussian peaks, which are subsequently subtracted to facilitate slope re-estimation prior to combining into a representative model (Donoghue, 2020); or (3) non-integer resampling prior to Fourier-based spectral decomposition, followed by taking the median of the auto-spectral distribution (Irregular Resampling Auto-Spectral Analysis [IRASA]) (Wen and Liu, 2016). Given the challenges of comparing raw exponents acquired when performing different tasks (Gao et al., 2017), we focus here only on characterising resting-state 1/*f* ^β^ during typical development and maturation. We also explore evidence surrounding the maturation of activity patterns in the temporal domain via the resting Hurst exponent (HE), a measure which captures the self-similarity, trending or “persistence” of activity patterns within windows of a timeseries. These persistent patterns/trends constitute long-range temporal correlations (Hardstone et al., 2012; Jannesari et al., 2020) between (often unknown) underlying sources, which can provide an informative functional connectivity marker. HEs are typically calculated via detrended fluctuation analysis (DFA)(Peng, 1994, Peng, 1995). Similar to AEs, HEs reflect scale(s) of self-similarity/power law structure(s) but do not by themselves offer granularity as to the underlying spiking characteristics (or associated frequency profile) of signal generators, therefore do not allow decomposition into frequencies commonly associated with excitatory or inhibitory neuronal population activity. HE (α) can be converted into PLE for both stationary (α = 0–1, *i.e.* representing a linear system governed mostly by a singular scaling behaviour) and non-stationary (α = 1–2 *i.e.* representing a non-linear, multi-fractal system governed by multiple scaling behaviours) cases (Eke, 2000, Hardstone, 2012). Given the dynamic nature of the brain’s activity, EEG data generally display persistent patterns of electrical activity (0.50<*HE*<1.00) which are non-stationary (HE>0.50) *i.e.* activity does not revert to a baseline state but is segregated and maintained in contextual functional states. To further synergise the 1/*f* literature here we also convert HE into AE, wherein AE=2*HE-1 (Schaefer, 2014), thus providing a comprehensive account of early developmental 1/*f* ^β^ changes.

This systematic review aims to explore how and when 1/*f* measures change in early human development, and where variability within early lifespan stages exists, thereby offering a more nuanced perspective of sensitive periods of neurodevelopment.

## 2 Methods

### 2.1 Eligibility criteria and selection process

We included observational or experimental studies containing resting-state (eyes open [EOR] or closed [ECR]) data for ten or more typically developing (TD) human participants with a mean-centred age less than 26.50 yrs (*i.e.* bordering into ‘emerging adulthood’, see Hochberg and Konner, 2020) who were not otherwise known to have been born premature or hold any clinical diagnoses (neurodevelopmental, neurodegenerative or neuropsychiatric). For subjects younger than 2 yrs (neonates and infants ), data collected during sleep or wake (including when observing videos or toys) were included. We included studies which referred to AE or slope, 1/*f* ^β^, HE, fractal dimension (to assess for HEs), PLE/spectral slopes, or AE/PLE estimation models (*e.g.* FOOOF/specparams/IRASA/sprintf/PaWNextra). Abstracts fitting these criteria were assessed as full-texts if an English-language text was available, including abstracts referring to an evoked paradigm or where sample or method details were omitted, so as to capture suitable studies containing resting-state data for TD individuals within the aforementioned age range. Articles focusing on non-human populations (*e.g.* animals, or simulations only), of an unsuitable format (preprints, reviews, theses, case reports, books, conference abstracts, and non-peer-reviewed material) or using measures other than scalp-based EEG (*e.g.* iEEG, sEEG or ECoG, MEG, TMS, tDCS) were excluded. Articles lacking measures of interest in the main/supplementary texts were excluded. In calculating the HE, the underlying scaling exponent (α) only deviates from 0.50 for short window sizes (Hardstone et al., 2012), hence the scaling range should be reported. Furthermore, we exclude papers not reporting or responding to requests for two or more key details (scaling range, epoch length, window size).

### 2.2 Search strategy and information sources

The systematic review was completed according to the PRISMA guidelines (Page et al., 2021) and pre-registered with the international Prospective Register of Systematic Reviews (PROSPERO Registration number: CRD42023363294). Relevant literature referred to the development/maturation of the 1/*f* ^β^ signal: 1/*f*, aperiodic exponent/slope and/or the HE (Hurst exponent*/slope or fractal, primarily measured via DFA or detrend* fluctuation analysis) across the early human lifespan (birth, newborn, neonat*, infan*, toddler*, child*, adolescent, teenager, young adult*, develop*, maturation*) using EEG (EEG or electroencephal*). Searches were performed across the following databases (with appropriate MESH headings and adjacency terms where permissible): Ovid-Embase, Ovid-PsycInfo, Ovid-Medline, Scopus and Web of Science, during March 2023. For an example search strategy, see Supplementary Material **I**. Backwards searching of included studies was also performed.

### 2.3 Selection process

Records were stored and de-duplicated in Endnote before being transferred to Rayyan for secondary deduplication and subsequent screening. Titles and abstracts were screened by author RAS, with a subset (20 %) reviewed independently by co-author DM and re-reviewed in cases of disagreement until a consensus was reached.

### 2.4 Data collection and data items

Article full texts were then screened by RAS and data pertaining to sample characteristics (age mean and SD, sample size, gender split) and 1/*f* data (AE/PLE/HE) were extracted from tables or figures of the main and/or supplementary texts, an associated repository or by contacting the authors directly.

### 2.5 Study risk of bias assessment

Risk of bias was assessed independently by co-authors RAS and CE using the Quality Assessment for Diverse Studies (QuADS) tool (Harrison et al., 2021), with the omission of item 12 (stakeholder involvement) due to a lack of relevance to the TD population. Rater scores (91.07 % agreement) were compared to ensure differences of less than 2 points (0.01 %, 6/504 cases). Differing cases were discussed, agreed and calibrated. For the assessment criteria and risk of bias results, see Supplementary Materials **II and III** respectively.

### 2.6 Synthesis methods

Few studies reported age correlations or other effect sizes (N=8) and given the ambiguity of raw AE effect size interpretation (Gao et al., 2017) and the absence of a comparison state uniform to all studies, a meta-analysis was not performed. Rather, we qualitatively synthesised findings across lifespan stages: infancy (0.01–2.00 yrs), toddlerhood (2.00–3.00 yrs), childhood (3.00–12.99 yrs), adolescence (13.00–19.99 yrs), young adulthood (20.00–26.00 yrs), spatial scale (global, regional, channel-wise), method (HE, PLE/AE) and condition (ECR/EOR).

## 3 Results

**Fig. 1:**
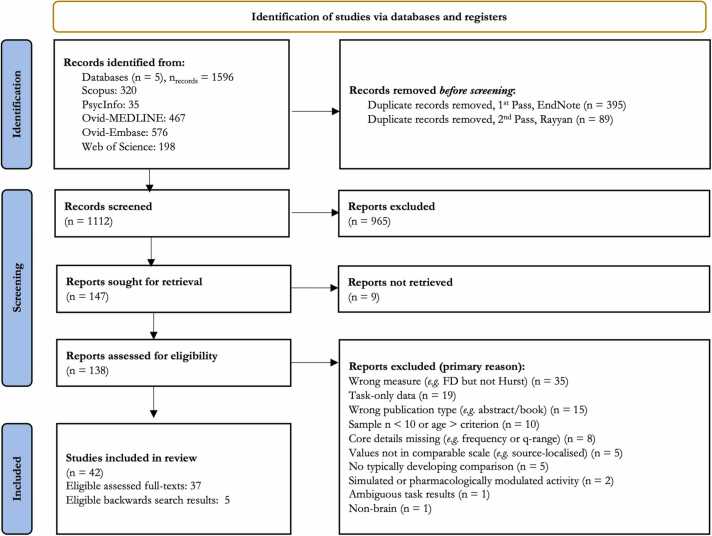
PRISMA flowchart for record screening. Backwards searching utilised based on citation title relevance of included texts to ensure sufficient article capture (N=5 relevant reports, see ‘Included’).

Our database searches yielded 1596 records. After de-duplication, we screened 1112 titles and abstracts. Nine full-texts sought for retrieval were unavailable, resulting in 138 retrieved full-texts, of which 37 were included (see Fig. 1). We identified a further five studies after searching for citations of the included studies as well as their reference lists. The characteristics of the included studies are shown in Table 1.

**Table 1:** Studies included in the review. Infancy (0.01–2.00 yrs), Toddlerhood (2.00–3.00 yrs), Childhood (3.00–12.99 yrs), Adolescence (13.00–19.99 yrs), Young adulthood [YA] (20.00–26.00 yrs). ‘ est ’ in the ‘Scale’ column denotes values are estimated from a plot. Measures include eyes open (EOR) and closed (EOR) rest, alongside other specified states. Measures from sub-samples in the ‘Original Measure’ column are referred to by ‘S’ whilst observed timepoints are denoted by ‘t’. Sample split by sex is given in ‘N (M, F)’, wherein unknown values are indicated by ‘?’. Data from supplementary sources (tables, figures) are denoted as ‘Supp.’ in the ‘Source’ column, with open-access data from the open science framework (OSF) marked and ‘Auth Corr.’ denoting author correspondence was required for additional information/data was absent from the published material. Sections and figures (‘Fig’) are marked where relevant. Studies with overlapping data are marked with the same superscript character (a,b respectively). In column ‘F’, ‘Y’ entries denote backward-search results. For technical details of measures, see Supplement IV.

**#**	**Study**	**Lifespan Stage (age, yrs)**	**Measure**	**Scale(s)**	**Original Measure**	**HE to AE**	**Measure**	**N (M, F)**	**Source**	**F**
1	Schaworonkow and Voytek (2021)	Infancy (0.10–0.56)	1/f (FOOOF)	Channelwise	S1: 1.74–3.22 (N = 20) S2: 1.74–2.95 (N = 20) S3: 1.79–2.25 (N = 20) S4: 1.94–2.98 (N = 5) S5: 1.46–2.76 (N = 3) S6: 1.88–2.63 (N = 2)		Baseline-wakeful reaching	22(10,12)	Methods, Auth Corr.,GitHub	
2	Karalunas et al., (2022)	Infancy (0.12±0.01) Adolescent (14.10±1.30)	1/f (FOOOF)	Global, Channelwise	Infant EOR (PEACH cohort): 2.21±0.28Adol EOR (1.85±0.28): ECR (1.98±0.26), EOR-ECR avg (1.91±0.28)Infant: 2.48±0.24 (Cz, EOR) Adol: 2.28±0.19 (Cz, EOR), 2.33±0.27 (Pz, ECR),2.29±0.19(EOR-ECR average)		EOR, ECR EOR-ECRavg	69 (36,33)152(85,67)	Auth Corr.	
3	Fransson et al., (2013)	Infancy (0.81, 0.75–0.85)	1/f (PLE)	Global, Regional, Channelwise	2.07±0.22		Natural Active/ Quiet Sleep	15(9,12)	Fig. 4	Y
4	Carter-Leno et al., (2022)	Infancy (0.90±0.05)	1/f (FOOOF)	Global, Regional, Channelwise	1.50±0.13 (non-social), 1.52±0.16 Fz: social (1.53±0.16), non-social (1.51±0.13)Cz: social (1.51±0.15), non-social (1.49±0.13)Pz: social (1.51±0.16), non-social (1.49±0.13)		∼EOR (social and non-social videos)	24(13,11)	Table 1, Fig. 4,Auth Corr.	
5	Roche et al., (2019)	Infancy (1.92–10.25)	1/f (PLE)	Global^est^, Regional	∼0.58		∼EOR (movie)	37(0,37)	Methods, Results	
6	Smith et al., (2021)	Infancy (med. 0.63, 0.43–0.82)	HE	Global	Delta[1–3 Hz]: ∼0.80 (A), 0.68(S) Theta[4–7 Hz]: ∼0.74(A), 0.68(S)Alpha[8–12 Hz]: ∼0.69(A), 0.68(S) Beta[13–30 Hz]: ∼0.88(A), 0.72(S)		Awake, Sleep	20(12,8)	Section 3.1, Fig. 6,Auth Corr.	
7	Smith et al., (2017)	Infancy (med. 0.58, 0.48–0.94)	HE	Global^s^	Delta[1–3 Hz]: ∼0.78Theta[4–7 Hz]: ∼0.70 Alpha[8–12 Hz]: ∼0.66 Beta[13–30 Hz]: ∼0.94		Awake∼(EOR)	21(?,?)	Fig. 5,Auth Corr.	
8	Cellier et al., (2021)	Toddler (N=5), Child (N=81), Adolescent (N=22), Young Adult (N=8)(2.95–24)	1/f (FOOOF)	Regional (Parietal-midline [P], Frontal-midline [F])	Toddler: [P] 1.45±0.23, [F] 1.32±0.54Children: [P] 1.23±0.25, [F] 1.34±0.22Adolescents: [P] 1.24±0.18, [F] 1.13±0.24Young Adults: [P] 1.14±0.12, [F] 1.11±0.09		EOR	116 (33,24,59 unlabelled)	Fig. 2, Sections 2.2, 3.1, Auth Corr.,OSF	
9	Houtman et al., (2021)	Toddler (2.92 [N=8], 3.92 [N=13]), Child (7–16[N=29])	1/f (FOOOF), HE	Global Channelwise	Infant-toddler (I) & child-adol (C): HE, 11–18 Hz: I: ∼0.655, C: ∼0.656Infant-toddler (I) & child-adol (C): AE, ∼1.11–1.60 (Hurst, 11–18 Hz): I: ∼0.63-.70), C: ∼0.64–0.74, 0.66±0.02		EOR	50 (28,22):Inf-Todd:21 (14,7)Child-Adol:29 (14,15)^a^	Figs. 3, 5Supp. Fig. 5	
10	Wilkinson and Nelson (2021)	Child (3.98±1.09, 2.67–6.67)	1/f(FOOOF)	Global Regional	1.19±0.12 Frontal: 1.26±0.13 Central: 1.33±0.14 Temporal: 1.11±0.15 Posterior: 1.07±0.32		EOR	12(12,0)	Methods, Results,Auth Corr.	
11	Robertson et al., (2019)	Child (5.65±1.23)	1/f (FOOOF)	Global Channelwise	1.51±0.32		EOR	50(36,14)	Table 1, Fig. 2A, B	
12	McSweeney et al., (2023)	Child (6.92±2.21)	1/f (FOOOF)	Global	EOR: 1.53±0.31 ECR: 1.77±0.28		EOR, ECR	502(230,272)	Section 3.2, Auth Corr.	
13	Ahmad, 2022, Arnett, 2022	Child (8.83±1.23)	1/f (FOOOF)	Global	1.77±0.15 (Median: 1.76)		EOR	29(19,10)^b^	Methods, Auth Corr.	
14	Arnett et al., (2022b)	Child (8.83±1.23)	1/f (FOOOF)	Global	1.77±0.15 (Median: 1.76, range: 0.22–2.30)		EOR	29(19,10)^b^	Methods,Auth Corr.	
15	Peisch and Arnett (2022)	Child (9.40±1.36)	1/f (FOOOF)	Global Regional	1.78±0.14 Anterior Frontal (AF): 1.79±0.14 Frontal (FR): 1.79±0.13 Central (CE): 1.75±0.15 Parietal (PR): 1.81±0.16 Occipital (OC): 1.77±0.22		EOR	29(19,10)^b^	Methods, Auth Corr.	
16	Hill et al., (2022)	Child (9.41±1.95)	1/f (FOOOF)	Global Regional (anterior [A], central [C], posterior [P])	EOR: 1.65±0.18 ECR: 1.81±0.16 EOR: A (1.64±0.19), C (1.69±0.19) P (1.68±0.20) ECR: A (1.81±0.17), C (1.85±0.16), P (1.84±0.18)		EOR, ECR	139 (72, 67)	Fig. 2,Auth Corr.	
17	Tröndle et al., (2022)	Child (N=153), Adolescent (N=34), Young Adult (N=3)(10.07±3.39, 5.02–21.67)	1/f (FOOOF)	Regional (Parieto-occipital)	1.89±0.36 (0.68–2.77) Child: 1.98±0.30 Adolescent: 1.58±0.37 Young adult: 1.12±0.04		ECR	190(104,86)	Methods,Auth Corr.,Fig. 3, App. 4, Supp. 2, 3	
18	Kwok et al., (2019)	Child 4 yrs (N=8), 5 yrs (N=14), 6 yrs (N=11),(5.60±?.??)	HE	Global, Channelwise^est^	Median: ∼0.09 (EOR) ∼0.06(ECR) Posterior electrodes: ∼0.09 EOR, ECR		EOR, ECR	33(?,?)	Fig. 6A-C	
19	Smit et al., (2011)	Child (5.27±0.19,6.79±0.19), Adolescent (16.06±0.5517.57±0.55), Young Adult (26.18±4.15)(5−50)	HE	Channelwise (12 channels)	Child Theta (P3 maxima): 0.77±0.09 (5 yrs), 0.76±0.07 (7 yrs) Child Alpha (O2 maxima): 0.70±0.09 (5 yrs), 0.71±0.08 (7 yrs) Child Beta: 0.64±0.09 (5 yrs, Fp2), 0.62±0.08 (7 yrs, F8) Adol Theta (Fp1 maxima): 0.72±0.06 (16 yrs), 0.72±0.06 (18 yrs) Adol Alpha (O1 maxima): 0.72±0.10 (16 yrs), 0.73±0.12 (18 yrs) Adol Beta: 0.64±0.09 (16 yrs), 0.66±0.11 (18 yrs) YA Theta (F3 maxima): 0.73±0.07 (25 yrs) YA Alpha (P4 maxima): 0.75±0.09 (25 yrs) YA Beta (O1 maxima): 0.67±0.10 (25 yrs)		ECR	5 yrs 366 7 yrs 378 16 yrs 426 18 yrs 387 25 yrs 396	Auth Corr. Methods,Fig. 3,Table 2	
20	Bruining et al., (2020)	Child (10.30±1.54)	HE	Global	0.66±0.04		ECR	29 (14,15)^a^	Supp. Table 1	Y
21	McSweeney et al., (2021)	Adolescent (12−17)	1/f (FOOOF)	Global (1–45 Hz)	t_1 (all subjects)_: EOR (1.21±0.30) t_1 (subjects with t1 & t2)_: EOR (1.21±0.30) t_1 (all subjects)_: ECR (1.33±0.27) t_1 (subjects with t1 & t2)_: ECR (1.21±0.30) t_2 (all subjects)_: EOR (1.10±0.26) t_2 (subjects with t1 & t2)_: EOR (1.11±0.27) t_2 (all subjects)_: ECR (1.16±0.25) t_2 (subjects with t1 & t2)_: ECR (1.17±0.26)		EOR, ECR	186(85,101)95 @t_1_, t_2_	Fig. 1B, Results	
22	Ostlund et al., (2021)	Adolescent (13.97±1.28)	1/f (FOOOF)	Global (2–50 Hz)	(EOR+ECR/2): 1.80±0.28 (0.92–2.57) EOR: 1.72±0.31 (0.88–2.49) ECR: 1.88±0.28 (0.90–2.65)		EOR, ECR	97(53,43)	Table 1	
23	Linkenkaer-Hansen et al., (2007)	Adolescent (16.50–19.50)	HE	Channelwise (Alpha, Beta)	Alpha: (0.70–0.74±0.08–0.11) Beta:(0.61–0.66±0.07–0.09)		ECR	390(196,194)	Table 1	
24	Gao et al., (2017)	Adolescent (18.30±2.80)	HE	Channelwise (Delta-Gamma)^est^	Alpha: ∼0.80 Beta: ∼0.70	0.600.40	ECR	15(15,0)	Fig. 2	
25	Donoghue et al., (2020)	Young Adult (19.56±1.90)	1/f (FOOOF)	Channelwise (Cz)	1.43±0.25		EOR	16(8,8)	Auth Corr., Results	
26	Linkenkaer-Hansen et al., (2001)	Young Adult (20−30)	1/f (PLE) HE	Global (Alpha [8–13 Hz]) 4-channel avg	PLE ECR: 0.36±0.17 PLE EOR: 0.51±0.12 HE ECR: 0.68±0.07 HE EOR: 0.70±0.04		EOR, ECR	10(9,1)	Results	Y
27	Muthukumaraswamy and Liley (2018)	Young Adult (23.00±??)	1/f (IRASA)	Global, Channelwise	β_lf_ 1.36(1.12–1.72) β_hf_ 1.48(1.18–1.81) β_lf_ frontal maxima: 1.72 β_hf_ central maxima: 1.81		ECR	17(17,0)	Methods,Supp. Fig 7	
28	Pathania et al., (2021)	Young Adult (20.88±2.24)	1/f (PLE) 1/f (FOOOF)	Global (FOOOF), Regional (FOOOF),	1.36±0.26 F(1.18±0.34), C(1.40±0.28), P(1.46±0.28), O(1.41±0.29)		EOR	59(19,40)	Auth Corr.	
29	Barry and de Blasio (2021)	Young Adult (21.20±3.80)	1/f PN Slope(PaWNextra)	Global Channelwise (30 channels)	EOR (session 1, 2 average): 1.07±0.33 ECR: 1.22±0.38 EOR: 0.41–1.50 (Fp1, Cz) ECR: 0.38–1.22 (Fp1, C4)		EOR, ECR	20(3,17)	Auth Corr.	
30	Merkin et al., (2023)	Young Adult (22.20±3.90,18–35)	1/f (FOOOF)	Global^est^ Regional	∼1–2.1∼ range 1.3–1.6 YA		ECR	85(37,48)	Sections 2.1, 3.1, Supp. S5	
31	Ke et al., (2022)	Young Adult (22.29±2.28)	1/f (FOOOF)	Global, Regional (Frontal, Central, Parietal, Occipital)	Global (1.84±0.34) Frontal (1.99±0.35) Central (1.84±0.34) Parietal (1.76±0.37) Occipital (1.67±0.52)		EOR	90(44,46)	Table 1, Auth Corr.	
32	Smit et al., (2013)	Young Adult (22.40, 21–25)	1/f (PLE)HE	Channelwise (Alpha [9–13 Hz]) CP3	Maxima (both): central midline, scalp ranges Hurst (0.70–0.80), 1/f (0.20–0.40) PLE = 0.43 HE = 0.66 (Range: 0.66–1.04)		EOR	39(11,28)	Fig. 1B/C, Auth Corr.	Y
33	Zsido et al., (2022)	Young Adult (22.48±3.79)	1/f (FOOOF)	Global^est^	∼1.40		ECR	31(?,?)	Methods	
34	Immink et al., (2021)	Young Adult (22.67±3.85)	1/f (IRASA)	Global	2.06±0.13 (range: 1.82–2.48)		ECR	45(22,23)	Section 3.1, Auth. Corr.	
35	Pathania et al., (2022)	Young Adult (23.29±3.47)	1/f (FOOOF)	Global (2–25 Hz), Regional	1.17±0.23 F(1.20±0.25), C(1.22±0.27), P(1.09±0.28), O(0.96±0.28)		ECR	21(11,10)	Section 4.1, Fig. 2B, Auth Corr.	
36	Cross et al., (2022)	Young Adult (25.00±7.13)	1/f (FOOOF, IRASA)	Global	*IRASA* ECR: 1.11±0.30 *IRASA* EOR: 1.08±0.31		EOR, ECR	35 (18,17)	Auth Corr.	
37	Nakao et al., (2019)	Young Adult (19.57±??18–21)	HE	Alpha [8–13 Hz]	FCz: 0.75±0.12 Min (T7, 0.74±0.12) Max (O1, 0.80±0.13)	0.50 (0.48–0.60)	ECR	23(11,12)	Fig. 5, Section 3.3, Auth Corr.	
38	Natarajan et al., (2004)	Young Adult (20.00±3.00)	HE	Global (1–50 Hz)	0.29±0.06	-0.42	ECR	30(15,15)	Table 1	
39	Liu et al., (2022)	Young Adult (20−30)	HE	Channelwise^est^ (Broadband)[0.5–120 Hz]	EOR∼0.80–0.82	EOR0.60–0.64	ECR, EOR	26(?,?)	Fig. 5	
40	Sleimen-Malkoun et al., (2015)	Young Adult (22.70±1.60, 18.80–25.10)	HE	Global (0.5–100 Hz)	1.69 Higher for posterior vs midline	2.38	ECR	31(17,14)	Fig. 4	Y
41	Irrmischer et al., (2018)	Young Adult (25.00±6.20)	HE	Global (Delta [1–4 Hz], Theta [4–8 Hz], Alpha [8–13 Hz], Beta [13–45 Hz])	ECR (N = 57) Theta: 0.66±0.01 Alpha: 0.71±0.01 Beta: 0.66 ± 0.01 EOR (N = 23) Theta: 0.69±0.02 Alpha: 0.75±0.02 Beta: 0.70 ± 0.01	ECR 0.32 0.42 0.32 EOR 0.38 0.50 0.40	EOR, ECR	57(22,35)	Results	
42	Bornas et al., (2013)	Young Adult (24.61±7.03)	HE	Regional (theta [3–7 Hz], alpha [8–13 Hz], broadband [1–40 Hz]): Central [C], Parietal [P], Occipital [O])	Theta C (0.75±0.07), P (0.76±0.07), O (0.74±0.07) Alpha C (0.76±0.07), P (0.80±0.08), O (0.85±0.10) Broadband C (0.85±0.07), P (0.86±0.06), O (0.88±0.06)	Theta 0.50,0.520.48 Alpha 0.52, 0.600.70 Broadband 0.70, 0.720.76	EOR, ECR average	56(20,36)	Table 1	

### 3.1 Risk of Bias

Across the 12 QuADS items examined, the performance of included studies was generally strong across all items with average scores exceeding 2 (scale 0–3, Supplementary Material **III**). Studies generally showed the weakest performance in terms of providing recruitment data, discussing study strengths and limitations and providing clearly defined research aims/hypotheses.

### 3.2 Narrative synthesis

Of the 42 included articles (N=3478 aggregated observations; 99 HEs+AEs/PLEs, 1097HEs, 2282 AEs/PLEs), seven included infants (5 AE/PLE, 2 HE), two included samples containing toddler cohorts (1 AE, 1 AE & HE), thirteen included children (9 AE, 3HE, 1 AE & HE), eight included adolescents (5 AE, 3 HE), and twenty-one included young adults (12 AE, 7 HE and 2 HE and PLE). Most studies analysed data in either EOR or ECR conditions, though two studies used EOR-ECR averages to increase the signal-noise ratio (SNR) (no statistical ECR-EOR differences were reported). The majority of 1/*f* ^β^ studies used the FOOOF package (22/42), and thus, for brevity, studies should be assumed to use FOOOF unless otherwise stated. Results are discussed as measured (*i.e.* HE as HE, not AE), with later discussion on the utility of value conversion (see also Table 1).

**Fig. 2:**
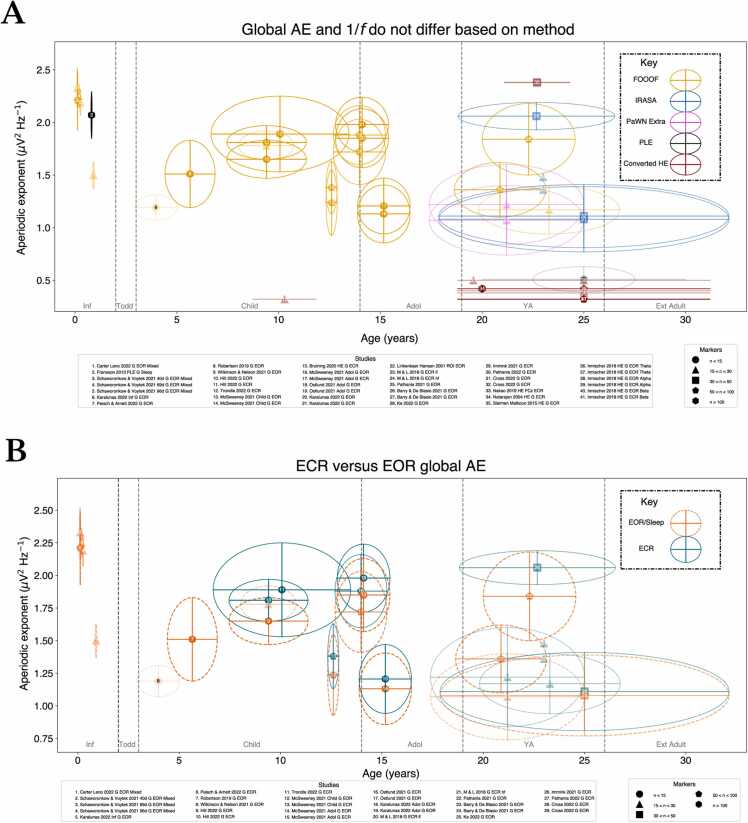
Consistency of the (A) global aperiodic exponent (μV^2^ Hz^−1^) across methods and (B) resting-state method for each lifespan stage. Studies are denoted beneath each plot, both figures include eyes open (EOR) and closed (ECR) rest; larger samples are encoded with higher alpha in each plot; see the marker legend for corresponding glyphs. Inf: Infancy, Todd: Toddlerhood, Child: Childhood, Adol: Adolescence, YA: Young adulthood, Ext Ad: Extended adulthood. Horizontal whiskers denote study age standard deviation (SD) whilst vertical whiskers denote 1/*f*^β^ SD. Study numbers (white, black) only differ to enhance readability. ‘lf’ and ‘hf’ denote low and high frequency slope estimation ranges. For visibility, only global AEs are shown, whilst converted HE may include regional measures as the only recording sites available.

**Fig. 3:**
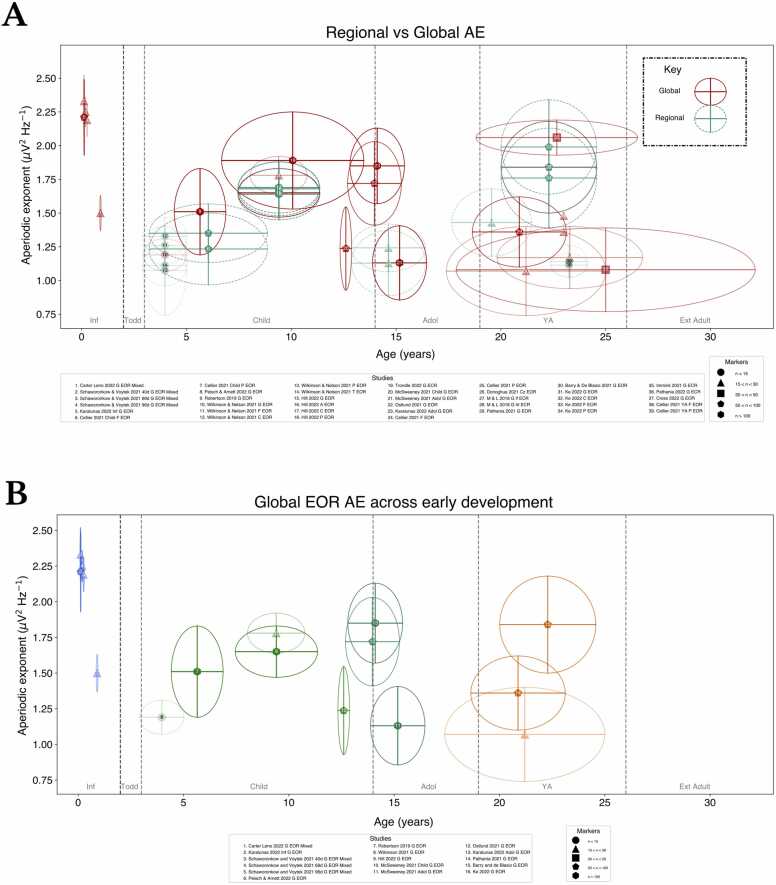
Consistency of the (A) global aperiodic exponent (μV^2^ Hz^−1^) across global and regional scales and (B) focusing explicitly on the global trend of AEs across lifespan stages. For studies where both EOR and ECR were available, only EOR was plotted, as to avoid excess overlap. (A) dotted lines indicate regional AEs, whilst solid lines denote global AEs. Alpha encoding as in Fig. 2. Study numbers (white, black) only differ to enhance readability. (B) Colourisation by lifespan stage from infancy-young adulthood. ‘lf’ and ‘hf’ denote low and high frequency slope estimation ranges.

Overall, the method employed to measure 1/*f* ^β^ only has a marked impact when comparing converted HE with measurements of AE/PLE, whilst comparisons of direct measures (*i.e.* measures not converted from HEs) show no difference between calculation methods (Fig. 2**A**). Focusing on direct AE measures, the global AE decreases from infancy to toddlerhood and remains within more confined AE ranges thereafter (Fig. 2**B**). However, the interpretation of this trajectory hinges on an accurate characterisation of AEs during infancy (via sufficiently powered studies), whereas currently, few studies exist. Further, there does not appear to be a difference between global versus regional AEs across the lifespan (Fig. 3**A**), evident also on a regional scale (**Supplement V**). Both ECR and EOR AEs display broad variability (Fig. 2**B**), particularly in young adulthood (YA), irrespective of study size. Following infancy, regional and global age-related changes generally overlap, with the highest (global) between-study variability observed in YA. These data suggest no differences between AE estimation method, resting-state paradigm, or the level of scale measurement (for most stages). Given the comparability of EOR and ECR, we plot results only for EOR where study data for both conditions is available.

**Fig. 4:**
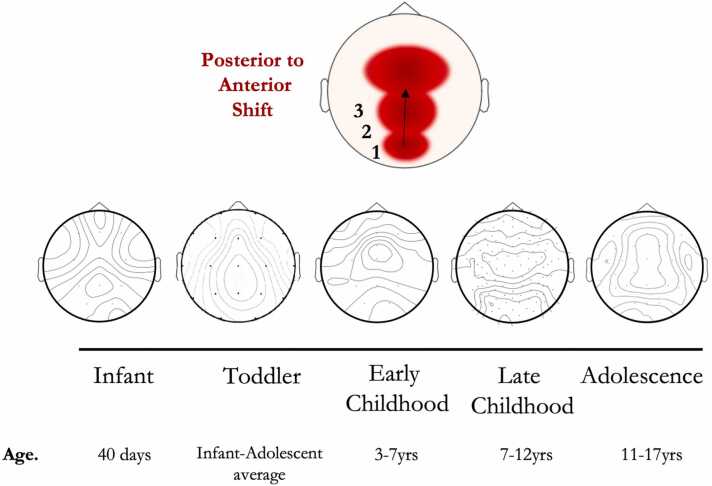
Illustrative regional maturation of the aperiodic exponent (μV^2^ Hz^−1^) with age. Due to limited access to study data for studies in each lifespan stage, topoplots have been generated from available eyes-open rest (EOR) data (references: #1, #9, #11, #15, #2 respectively). For the toddler topoplot, transparency edits to the corresponding published topoplot were made as the data were not publicly available or supplied on request.

In EOR, we observe age-related AE stabilisation following infancy, with the centre of this trajectory in line with the infant AE estimates of Carter Leno et al., (2022)(Fig. 3**B**). In summary, there is insufficient evidence in infancy-toddlerhood to validate exponential AE decay, and from childhood onwards AEs seem to vary (partly due to the broad spread of ages in individual studies, as reflected in the age SDs). Whether consistent AE decreases occur from infancy to toddlerhood is likely to be better revealed by studying data at the individual level, dissecting both within- and between-study variability with greater precision. This includes exploring the impact of parameter decisions, such as the number of peaks fit and peak height which affect slope estimation and therefore AE estimates. Notably, the topography of the AE changes with age (Fig. 4) with AE maxima shifting from posterior foci during infancy (and early toddlerhood) to the midline with continued development. Comparatively, changes in the HE across early development are equally subtle, with evidence from the majority of included studies illustrating that HEs vary by < 0.10 for any given band across the early life span.

### 3.3 Hurst exponent: Infancy-young adulthood

For the HE synthesis, twenty-four studies were included: seven containing infants, one containing toddlers, four containing children, three containing adolescents and nine containing young adults. Infants displayed persistent neural activity patterns (HE>0.50) from delta-beta bands (1–30 Hz), during wake (Smith et al., 2017) and sleep (Smith et al., 2021). Whilst no studies explicitly examined HE maturation in infancy or toddlerhood, one study used a sample containing toddlers. Houtman et al. (2021) showed that in an infant-toddler sample, HEs vary from ∼0.65–0.68 across the scalp (with no statistical difference observed between toddlers and children). In childhood, studies collectively showed similar HE in older children, with HE also >0.50: Bruining et al. (2020) showed global HE of 0.66±0.04 during ECR in older children (the same sample used in Houtman’s work). Moreover, in a study from ages 5–71 yrs (5–7, 16–18 yrs longitudinally), Smit et al. (2011) identified age-related changes in alpha (5–18 yrs occipital maxima, 25 yrs: parietal maxima) and beta band HEs (5, 7 yrs frontal maxima, 16–50 yrs: occipital maxima). By contrast, the theta band HE of Smit et al. (2011) are stable and parietal dominant from childhood (5 yrs) until YA (25 yrs) before switching to frontal dominant in adolescence. Conversely, Kwok et al. (2019) observed anti-persistent trends (HE<0.50) for global alpha band HE (EOR: ∼0.09, ECR, ∼0.06) and identified ECR-EOR differences unrelated to age. HEs remain consistent throughout adolescence, with ECR alpha and beta band HE (Linkenkaer-Hansen, 2007) similar across studies (Gao et al. (2017). In YA, Smit et al. (2013) identify EOR alpha band HE maxima in the central midline consistent with other ECR and EOR studies (Linkenkaer-Hansen, 2001). Moreover, other EOR studies illustrate increasing HE with age; both Nakao et al. (2019) and Liu et al. (2022) reported consistent ECR alpha band HE across the scalp. However, Natarajan et al. (2004) report considerably lower global HE (0.29 vs ≥0.70–0.80 in other studies), and Sleimen-Malkoun et al. (2015) identify a broadband global HE of 1.69, suggesting non-stationary (variation unrestricted to a singular mean/setpoint) with occipital maxima and frontal minima. Overall, studies continue to demonstrate posterior (occipital) HE maxima for the alpha band (Irrmischer, 2018, Bornas, 2013) consistent with prior power-based studies. In addition, Irrmischer et al. (2018) showed that for theta and beta bands, global EOR HE exceed ECR HE. Overall, the HE lifespan trend entails mostly subtle increases in HE with age, differing depending on the band examined and falling within a range of 0.60–0.80 (non-stationary and persistent).

### 3.4 Aperiodic/power law exponents: Infancy-Young adulthood

For the AE/PLE synthesis, thirty-six studies were included: five containing infants, two containing toddlers, ten containing children, five containing adolescents and fourteen containing young adults. Infant AE (typically >2.00) was higher than in any other lifespan stage for the included studies and appeared to decrease throughout infancy. For instance, Schaworonkow and Voytek (2021) described global AE decreases from 40 to 134 postnatal days with posterior maxima (40–70days: 3.21, 70–96days: 2.95, 96–134 days: 2.75). In younger infants (0.12 vs 0.81 yrs), Karalunas et al. (2022) observed EOR-ECR AE averages that were maximal in the midline (2.48, Cz). Conversely, Carter Leno et al. (2022) studied 10-month-old infants and identified global AE of 1.50, with no significant regional AE differences nor age effects. By contrast, the evidence from PLE studies shows much lower 1/*f* estimates; during movie-watching, infant global PLE was ∼0.58 for Roche et al. (2019), considerably below global PLE observed by Fransson et al. (2013) (2.07, occipital cortex).

The largest gap in the developing AE literature sits in toddlerhood; toddler AE are the least characterised of the studied lifespan stages, with only one study evident (Houtman, 2021), which described AE that were maximal in the midline (∼1.50–1.60). Additional insights were gained from the toddler sub-cohort (N=5) of Cellier et al. (2021) wherein steeper posterior (1.27–1.83) than frontal (0.47–1.81) AE were observed. Moreover, Cellier *et al.,* found that AEs significantly decreased with age across their full cohort (3–24 yrs) from toddlerhood through young adulthood (*r* = −0.36).

In comparison with other lifespan stages, AE and HE have been best characterised in childhood. Childhood studies recruited TD (McSweeney, 2023, Tröndle, 2022, Cellier, 2021) and case-control child cohorts for comparison with neurodevelopmental conditions including ADHD (Peisch and Arnett, 2022); Arnett, *et al.,* 2022a, b) and Fragile X syndrome (Wilkinson and Nelson, 2021). Childhood AE studies show higher AE than in infancy-toddlerhood, shifting from negative linear AE decay to a positive trend from early to late childhood. Studies in overlapping ages for early (Wilkinson and Nelson, 2021, Robertson, 2019, Peisch and Arnett, 2022, Hill, 2022) and late childhood (Tröndle, 2022, Ostlund, 2021, McSweeney, 2021) are generally in agreement in terms of both the direction and range of AE, for both ECR and EOR (Fig. 2**B**) and regional versus global (Fig. 3**A**) respectively. This was also consistent with figure estimates for studies where data could not be directly obtained (Houtman et al., 2021). Two studies provided statistical evidence of a negative age-related AE trend; firstly by Peisch and Arnett (2022) in younger children (*r* =-.30, consistent with previous overlapping work: Arnett *et al.,* 2022a, b), and secondly by McSweeney et al. (2023) in older children where a quadratic age-related AE decrease was observed, and ECR AE (1.77) significantly exceeded EOR (1.53) (a trend shown in other studies across the early lifespan, see Fig. 2**B**).

Two studies in childhood which have both AE data for ECR and EOR demonstrated these conditions to be comparable (Hill, 2022, Ostlund, 2021), alongside single-condition data (typically EOR) from other studies in this stage (Fig. 2**B**). Equally, two studies with AE measures for both global and regional scales highlighted comparability across scales (Wilkinson and Nelson, 2021, Hill, 2022), with similar trajectories evident based on combined data from other studies as in Fig. 3**A** (Robertson, 2019, Ostlund, 2021, Peisch and Arnett, 2022, Tröndle, 2022, McSweeney, 2021). Significant relationships between AE for both scales have also been reported for EOR but not ECR (Hill et al., 2022): [global] *r* = −.24, [regional] anterior: *r* = −0.28, central: −0.24, posterior: −0.35). Topographically, AE maxima in late childhood seem to be parietal dominant (Peisch and Arnett, 2022, Tröndle, 2022).

In adolescence, three studies provided quantitative evidence for age-related AE decreases (Ostlund, 2021, McSweeney, 2021, Karalunas, 2022), with additional support for this decreasing trajectory in sub-cohort data from Cellier et al. (2021). Age-related decreases are observed for both ECR and EOR (Ostlund, 2021, McSweeney, 2021), with lower AE observed in females, and faster age-related flattening observed in males (McSweeney, 2021). Given the collinearity between EOR and ECR, some authors opted to average across conditions (Ostlund et al., 2021). Topographic data was only available from one study (Karalunas et al., 2022), highlighting AE maxima in the central midline and lateral electrodes (extending more frontally and laterally in higher density caps), with lower adolescent AE than in the study’s infant sample.

A more complex trend is observed during YA, with divergent lines of evidence suggesting an increased versus decreased age effect when taken as a whole. Early YA resting-state PLE studies report considerably lower estimates than studies leveraging methods accommodating for oscillatory peaks to derive AE. For example, Smit, Linkenkaer-Hansen and de Geus (Smit et al., 2013) identify EOR PLE maxima in the central midline (0.20–0.40) whilst Muthukumaraswamy and Liley (Muthukumaraswamy and Liley, 2018) use IRASA to account for knees in the spectra by modelling multiple slopes, identifying global AE of 1.36 (β_1_:0.1–2.5 Hz, frontal maxima) and 1.48 (β_2_:20–100 Hz, central maxima) respectively. AE studies cluster between 1.30 and 1.60, similar to the range described by Merkin et al., (Merkin et al., 2023), irrespective of whether FOOOF (Donoghue, 2020, Pathania, 2021, Pathania, 2022, Zsido, 2022, Cross, 2022), IRASA (Muthukumaraswamy and Liley, 2018) or other methods (Barry and De Blasio, 2021) are utilised, and with similar patterns for ECR and EOR, though ECR AE remains higher. Two exceptions to this are noted (Ke, 2022, Immink, 2021), with one of these (Immink et al., 2021) identifying ECR AE estimates falling within the tentative infant AE range (>2.00). Merkin *et al.,* also noted that regional (but not global) age-related AE changes were significant when accounting for peak parameters and goodness of fit and did not differ by region.

In YA, the magnitude of ECR AE exceed that of EOR AE (Pathania, 2022, Cross, 2022, Barry and De Blasio, 2021), and topographical maxima centre around the central and frontal regions (Pathania, 2022, Barry and De Blasio, 2021), with an indication that this is more commonly frontal dominant (Ke et al., 2022). The differences in regional AE are smaller than in other lifespan stages, thus differences between these maxima (*e.g.* parietal - (Pathania et al., 2021) vs occipital - (Pathania et al., 2022) are unlikely to reflect biological differences.

## 4 Discussion

In this systematic review, we aimed to explore how and when EEG derived 1/*f* measures change in early human development, and where variability within early lifespan stages exists. We found that AE and HE age-related changes have complex developmental patterns; (1) HE consistently exceeded 0.50 across development, suggesting persistent and non-stationary signals throughout the early lifespan (2) provisional evidence suggests AEs decrease throughout infancy (*i.e.* an increased excitation:inhibition ratio) prior to the AE varying within confined ranges across subsequent development, (3) this pattern is generally consistent across AE methods, (4) the magnitude of ECR AEs exceed that of EOR AEs throughout early development (with overlapping trends observed), (5) heterogenous post-infancy AE changes do not differ between global or regional scales and (6) a posterior-anterior shift in maximal AE occurs from infancy through young adulthood.

### 4.1 Further evidence is required to determine age-related AE trends

Despite the influence of narrowband oscillations on slope fitting and exponent estimation, PLEs show age-related decreases (Waschke et al., 2017). We find that AE changes non-linearly from childhood onwards, a finding in line with large child AE datasets in both the EEG (Cellier, 2021, McSweeney, 2023) and MEG (Thuwal et al., 2021) literature. However, several other EEG studies fail to identify global (Merkin, 2023) or regional age-effects (Hill et al., 2022). As recent evidence suggests that the balance of E:I in early infancy may have key implications for brain development and function across the lifespan, infant AEs can provide an important early non-invasive marker of the integrity of functional brain activity. However, comparison of infant AE with AE in later life is likely to be affected not only by changes in neural activity across development, but also by non-neural changes to anatomy, including developmental changes to skull thickness, and changes in CSF volume, effects which will collectively impact the conductive properties of the skull in combination with progressive closure of the cranial sutures and fontanelles (posterior, anterior). As a result, this will impact AE estimation based on observed PSDs measured at the scalp. Currently, the provisional evidence available shows age-related decreases in global AE from the first several weeks after birth in term-born infants, however, there are significant gaps in the literature, particularly in mid and late infancy. Recently, Rico-Picó et al. (2023) identified early decreases in global AE (6–9 months) and flattening thereafter (9–18 months), a finding also observed by Brandes-Aitken et al. (2023). High AE in early infancy may reflect that a larger proportion of early infant EEG spectral power is concentrated in lower frequency ranges, with relatively lower power at higher frequencies (Marshall *et al.,* 2002; Saby and Marshall, 2012), leading to a steep spectral slope. The precise biology underlying these differences across this period is unclear but may represent regionally-varying maturational increases in glutamatergic receptor density (Johnston, 1995, Behuet, 2019) and glutamate (Kreis et al., 2002) and GABA concentration (Laurie et al., 1992, Turgeon and Albin, 1994, Kreis, 2002, Pinto, 2010, Xu, 2011, Behuet, 2019) which co-occur with the evolution of local circuitry and the rapid establishment of long-range connectivity. Importantly, the observed AE maturational trends appear robust regardless of the behavioural state of the infant during data collection (Schaworonkow and Voytek, 2021, Karalunas, 2022). However, we acknowledge that this area requires further systematic study as a combination of state, data quality and preprocessing approaches could have influenced the findings.

In toddlerhood, AE are less well characterised, which impedes the interpretation of a qualitative ‘trajectory’ of AE development thereafter (particularly given the complex patterns of AE variability observed in childhood). A preprint by Wilkinson et al., (Wilkinson et al., 2023) partially addresses this toddler AE gap, charting resting AE from 2 to 44 months, highlighting considerably flatter spectra than we observe here, with AE rising from 1.00 to 1.20 (0–1200 days) and age-sex interactions being evident. Evidence from this work suggests that AE increases persist through infancy and toddlerhood. Similarly, Witteveen et al., (Witteveen et al., 2023) identified progressive PLE slope increases in infancy and term-preterm PLE differences, though as previously discussed, PLEs do not account for the effect of oscillations on the 1/*f* slope so may differ from AE. Physiologically, increased postnatal AEs (higher inhibition/lower excitation) are in keeping with the axiom that postnatal GABA-related activity shifts from excitatory (depolarising) to inhibitory (hyperpolarising) postnatally due to changes in intracellular chloride concentrations (Ben-Ari, 2007, Kirmse, 2015, Ben-Ari and Cherubini, 2022). However, as this GABA shift occurs in immature neurons, E:I balance later tilts towards excitation (during or following late infancy) as circuits and networks mature and glutamate signalling predominates. Whether net excitation or inhibition initially dominates activity in the developing brain is frequently disputed: evidence from rodent (postnatal days 2–12) and newborn infant frontal EEG (35–46 postmenstrual weeks) 1/*f* data show higher AEs are observed with increasing age (Chini et al., 2022), possibly due to a more protracted integration of interneurons (relative to pyramidal neurons) into emerging circuits. Rodent data suggests that cortical GABAergic neuron cell fraction does not appear to change from early embryonic development until adulthood (Sahara et al., 2012), suggesting that changes in connectivity and glutamatergic cell density may be more focal influences of developing E:I balance. From childhood to young adulthood an age-related decrease in EEG AE in the DLPFC has been observed which was associated with glutamate but not GABA MRS measurements (McKeon et al., 2024), coinciding with evidence surrounding GABA concentrations stabilising in early life (Sahara et al., 2012).

Furthermore, there is currently no consensus on whether at the earliest point in infant development E:I balance tilts more towards excitation or inhibition, as AE coverage in this window is limited. Wilkinson et al. (Wilkinson et al., 2023)’s data suggests that all regional AEs but temporal AEs increase during infancy, whilst temporal AE decreases prior to a nadir ∼400 days, before increasing. Overall, these data and our findings agree that infant AE maxima are in the posterior channels overlaying occipital regions. However, our findings differ as to the direction of expected regional/global AE. Whilst beta frequency range peaks (10–20 Hz) in Wilkinson’s data may have affected AE estimation, the authors performed comprehensive model fit screening. They modified slope fitting functions in order to accommodate peaks, whereas modelling of knees or multiple slopes as in other work (Shuffrey et al., 2022) may have produced different findings. Rico Pico *et al.,* (2023) identified similar beta peaks which they attributed to muscle artefact and thus truncated their frequency spectrum: it would be beneficial to see how such the same truncation for the Wilkinson et al., (Wilkinson et al., 2023) data might affect AE estimates. A small number of other studies in this review parameterised a frequency range as widely as Wilkinson et al., (Wilkinson et al., 2023) using FOOOF (Robertson, 2019), Arnett *et al.,* 2022a,b, Wilkinson *et al.,* 2021, (Ostlund et al., 2021) with the majority parameterising at or below 40 Hz. Spectral widths are likely to vary developmentally (see Supplementary Material **IV** for the range variability between datasets, *e.g.* 1–10 Hz, 1–25 Hz, 1–40 Hz), with greater higher-frequency contamination in younger subjects owing to movement and muscle artefacts, which are not readily mitigated through instructions (*e.g.* for infants and toddlers). Moreover, the inclusion of higher frequencies (assuming such noise *is* mitigated and noise harmonics do not remain in the data) adds a source of largely excitatory contributions, and thus can affect E:I balance, and consequently AE estimation. Whilst observed differences between infants-toddlers and those of later ages are likely not simply due to such discrepancies in higher frequencies (as their power contributions decrease exponentially), this is nontrivial. Many groups do validate whether AE changes depending on spectral widths (*e.g* (Schaworonkow and Voytek, 2021)), but generally, this is rarely explicitly stated or evidenced in many publications. However, differences in electrical impedance and data quality during recordings and decisions made during pre- and post-processing leading up to AE estimation can all impact the estimate observed.

Other factors will influence AE estimation, with varying degrees of impact, including relatively minor influences such as the choice of power estimator across frequencies (*i.e.* Welch vs multitaper) to larger influences such as choices of filters during preprocessing, the parameterisation of inflection points in the spectrum and the number of peaks (and specification of their associated height and width) to fit using a given model. Ultimately, the validity of AE is entirely dependent on this fit being optimal. Whilst some of these decisions can be literature-led based on similar age ranges, there is no substitute for assessing (a) the quality of data, including its’ SNR, (b) visualising data directly to inform these decisions around modelling spectral ‘’knees’ and oscillations and (c) rejecting poor model fits, so that whether relating individual channel AE or global AE to a measure of interest, estimates are as meaningful as possible. Overall, future AE research should seek to provide robust estimates of AE, most crucially, as we have shown, during the neonatal period, identifying whether age-related decreases occur from the beginning of the neonatal period and continue through to late infancy. One approach to address this involves visualising pooled individual-level data across studies to get an accurate consensus of how AE varies within given stages (*e.g.* infancy) and, consequently, how variable it becomes across lifespan stages into adulthood. Importantly, the results of the group-level analysis reported here suggest that such an analysis should consider differing methodologies and model-fitting parameters to make robust comparisons.

### 4.2 Comparable AE results across methods

In contrast to early infancy, synthesising AE patterns across methods in subsequent childhood suggest that AE estimation methods are broadly comparable (except for converted HE): in YA in particular, FOOOF, IRASA, PawWNextra and PLE estimates overlap from 0.77 to 1.93. HEs differ in that whilst they inform us about the temporal persistence of EEG activity patterns (revealing when patterns become conserved across time), HEs are agnostic to the direction of change and tilting of the E:I balance spectra and thus only partially explain how E:I shapes evolving functional circuits/networks. Moreover, converted HE show substantial differences vs AE measured directly, likely as a result of: (1) many HE being characterised via DFA based on amplitude envelopes of specific bands (particularly papers from >2012), (2) time domain effects occurring due to reduced sampling windows and recording lengths, (3) data self-similarity which must be verified in source data for DFA and (4) conversion assuming DFA scaling exponents (⍺) are Gaussian (*i.e.* >0.5 or < 0.5) and not Brownian (∼0.5)(Eke et al., 2002). Finally, (5) the HE does not accommodate for oscillatory influence, and thus similar to PLEs, converted AE will provide potential over- or underestimates of the true AE, potentially providing physiologically implausible estimates akin to what has been described in the frequency spectra literature (Barry and De Blasio, 2021). Across the lifespan, HE studies consistently show persistent (0.50<HE<1.00), non-stationary (HE>0.50) patterns in each lifespan stage, reminiscent of sustained processing during measurement, and notably, of properties of a system with memory whose signal is exhibiting positive correlations over time (Hardstone *et al.*, 2012). Only Sleimen-Malkoun et al., (Sleimen-Malkoun et al., 2015)’s study suggested non-stationarity (1>⍺>2, therefore HE=⍺-1) in the EEG during rest. It is, however, worth noting that detecting developmental changes in HEs requires both large subject samples and (noise-free) long epochs (Berthouze et al., 2010) in order to characterise temporal correlations at multiple scales. This is all the more poignant given the individual variation in long-range temporal correlations within and across subjects (Linkenkaer-Hansen et al., 2007). Whilst the HE has been studied more deeply in adults, larger AE studies tend to focus on childhood. The HE studies captured by this systematic review were generally monofractal (a single scaling behaviour describes the trend) or equivalent multifractal measures (H(2)), though recent studies have tended to focus on multifractal EEG dynamics (wherein multiple scaling behaviours within a given temporal window are evident), potentially offering insights into more complex non-stationary scaling behaviours in EEG data (Zorick and Mandelkern, 2013); these methods thereby index E:I proxies at multiple temporal scales, akin to estimating 1/*f* slopes across multiple frequency ranges.

### 4.3 ECR consistently exceeds EOR AE

When focusing on AEs specifically, there was consistent evidence that the magnitude of ECR AEs exceeded that of EOR AEs across the lifespan, indicating there is, in fact, a difference between the underlying governing of neural activity in response to cues related to keeping the eyes open or closed. Furthermore, experimental instructions could also influence the net neural activity that comprises the observed PSDs and resultant estimation of AEs. Whilst this example is illustrative for differences in a seemingly benign case of “resting-state”, one can consider what more value-laden or association-rich wording differences in instruction may make under task conditions. Childhood studies showed marginal AE increases in later (Hill, 2022, Tröndle, 2022, McSweeney, 2021) versus earlier (Robertson, 2019, Wilkinson and Nelson, 2021) childhood (for both EOR/ECR), although this may reflect greater between-dataset variability rather than genuine AE increases preceding a prolonged age-related decline. Across early development, the greater magnitude of ECR vs EOR AE is driven not only by posterior-dominant alpha band activity (Wilson et al., 2022) but also activity in other frequency bands (Barry et al., 2007). Whilst some authors using other methods report FOOOF AEs are greater in EOR than in ECR, such as SPRiNT (Wilson et al., 2022), our findings consistently show that studies using FOOOF, IRASA and PaWNextra find ECR AE to exceed that of EOR. Moreover, both EOR and ECR AEs follow similar trajectories suggesting these ‘resting’ E:I processes mature in similar ways, consistent across both regional and global scales.

### 4.4 Regional vs Global AE

For the most part, global AE magnitude exceeds that of regional AEs (Fig. 3**A**), likely owing to the average rate of AE decrease across the scalp remaining constant across development whilst regional AE differs (as maturing regions shift developmentally). For example, regional changes in AE are apparent in early childhood, but equilibrate before YA and thus the neurobiological changes underlying AE changes during mature ageing are likely physiologically distinct from those in the earlier lifespan (Merkin et al., 2023). For example, using simultaneous EEG-fMRI during EOR, Jacob et al., (Jacob et al., 2021) identified posterior parietal AE maxima (∼1.60) in adults, with global average AE (1.49) being associated with decreases in frontal and increases in cerebellar, insular and cingulate blood-oxygen-level-dependent fMRI activity. In later life, Aggarwal and Ray (2023) identify there are no age-related MEG AE differences in younger versus older adults up to 50 Hz, but from 64 to 140 Hz AE decreases and from 230 to 430 Hz increases (higher inhibition), collectively suggesting that suPathabtle GABAergic changes may occur in later life outside of spectral ranges accessible to EEG. Moreover, whilst AE maturation may taper in earlier development, AE development is not static thereafter. Differences between mid and older adults were evident in posterior channels, similar to what is observed in early development, suggesting that network hubs established in infancy are also the last to change during later ageing. However, it is worth considering that when producing regional estimates to inform network maturation, selecting spatially neighbouring high SNR channels is vital (Linkenkaer-Hansen et al., 2001). Therefore, whether estimating regionally or globally, researchers should utilise model fit statistics to ensure adequate representation of underlying neural data. Currently, only a minority of studies report model fits and fewer still include fits as covariates. Given the need to systematically validate lifespan AE, we consider model reporting to be vital to ensuring accurate characterisation of developmental trajectories.

As to whether global versus regional AE are of greater utility, this will ultimately depend on the question under examination. For example, if the focal question centres upon sensorimotor development in infancy, then examining AE maturation within a region of interest comprising a subset of central electrodes constitutes a sensible approach. Alternatively, a researcher may be examining a phenotype in late childhood wherein no *a priori* brain source is implicated (or equally, where a network of spatially distributed sources is implicated) and thus utilisation of a global AE metric would be more appropriate.

### 4.5 Regional AE maxima shift across typical development

Topographical E:I maxima by definition relate to spectra with lower E:I balance (steeper AE spectra) relative to the rest of the brain, which in the absence of a stimulus (endogenous or exogenous) may suggest ongoing regional maturation (as opposed to flatter spectra and greater neural “noise” in ageing and pathology (Dave et al., 2018, Pertermann, 2019), or regions involved in networks which are more selectively held at baseline during conditions of rest. Understanding where AE are maximal (and neural noise minimal) provides insights into which regions are potentially undergoing maturational changes, which must first be characterised in TD to provide a referential maturational trajectory. We find that typical AE maturation displays a posterior-to-anterior shift in ageing (PASA), similar to the fMRI literature, with age-related reductions in occipital activity concomitant with increasing frontal activity (Davis, 2008, McCarthy et al., 2014). In longitudinal infant data at 6, 9 and 16 months, Rico-Picó et al. (2023) show AE decreases more slowly in occipital (maximal) and frontal versus parietal and central areas. These AE changes temporally coincide with white matter maturation and ongoing activity integration in the toddler as sensorimotor skills emerge (Hagmann et al., 2010). From childhood to late adolescence, the difference between posterior and anterior AE seems to grow with age across both sleep and wake, being the strongest in the second stage of sleep (Favaro et al., 2023). fMRI FC maturation at this point in development follows a sensorimotor-association gradient where primary sensory maturation precedes that of frontal executive and association areas (Sydnor et al., 2023). Overall, AEs demonstrate PASA in line with prior neuroimaging evidence, and accordingly, centro-frontal regions seem to be the maturational ‘endpoint’ for early AE development (with the lowest neural noise), perhaps supporting an increasing processing requirement for cognitive function.

### 4.6 Limitations and future work

This review highlights the complexity of characterising group-level age-related AE changes across studies, methods, and spatial scales. Given the heterogeneity in AE estimates, AE must be estimated on relatively noise-free data (minimal evidence of physiological/non-physiological artefacts including eye movements, electrode bridging, line noise, cardiac and respiratory signals or sweat-induced artefacts). Simulation work suggests SNR>2 are appropriate for determining HEs (Linkenkaer-Hansen et al., 2007). SNR may also be influenced by equipment selection, particularly for acquisitions with reduced channels, poorer contact quality and/or more flexible sensors, designed for ‘active’ paradigms (see Grummett et al., 2015). There are further influences due to data constraints and processing decisions, including the effect of window length on smoothing, affecting peak estimates. Data reference schemes will also affect PSD and AE estimates (Gao et al., 2017), for which most included studies used average referencing (see Table 1 and Supplementary Material **IV**). Equally, filtering decisions affect the frequency range available for exponent estimation; as others have shown, estimations on lower versus higher frequency slopes differ (Shuffrey, 2022, Muthukumaraswamy and Liley, 2018) and may have different physiological interpretations in the contexts of neurodevelopment and pathophysiology. In addition, motion is generally unavoidable in infants and children, who may not tolerate prolonged recording periods, hindering attempts to use epoch averaging to increase SNR.

AE estimation methods must, therefore, be valid for the applied dataset(s) and comparable with prior studies. For instance, when comparing FOOOF and IRASA results, a consideration is that IRASA evaluates spectral ranges beyond the fitted range in order to compute median AE using resampling factors (Gerster et al., 2022). Therefore, comparing results by exact frequency mapping results in evaluating upper or lower limit ranges which may be affected by filtering or noise, thus biasing AE estimation. Fortunately, IRASA and FOOOF AE estimates in this review overlap heavily, but this is nonetheless a consideration. Moreover, Gyurkovics et al. (2021) highlight that neural variability as captured by the 1/*f* ^β^ may differ between age groups, and spectra calculated from longer epochs (or averages) may be optimal for FOOOF as shorter single-trial spectrum models can overfit noise (due to the number of free parameters). For specific limitations and strengths of either method, see Gerster et al. (2022). Other tools for parameterising the AE have been introduced recently, but these have yet to be applied to resting scalp EEG (Seymour et al., 2022). Beyond methodological choices, AEs may also change based on genotype, task paradigm, and cognitive state (He, 2014, Voytek and Kramer, 2015, Donoghue, 2020), hence the focus on quasi-resting states and typical development in this review. The reviewed literature discussed provided (predominantly) cross-sectional measures of AE across development. However, the AE varies both statically across (*e.g.* 0.68–2.77 in (Tröndle et al., 2022) and dynamically within individuals (during recordings), especially in task-specific contexts. For example, Wilson et al. (2022) show that the AE varies over time in the resting-state, with YA AEs predictive of subject state (EOR vs ECR). Ultimately, more complex modelling may be required to evaluate how AE variation within-individual differs from results observed across individuals. Moreover, we suggest that the pooling of individual datapoints from constituent studies and a quantitative analysis thereof may better distinguish how age (and sex) influence biological AE changes across the lifespan.

Whilst individuals in our included studies may go on to attain diagnoses for one or more phenotypes, we worked from the basis of these individuals being TD at the point of measurement and from the published data. As more TD AE data becomes openly shared and built into trajectory models for AE maturation, and comparative non-TD trajectories characterised, future studies will be able to delineate critical junctures for deviation in a phenotype-specific or individual-specific manner to further our understanding of how altered AE is associated with non-TD phenotypes. Whether or not AE has sensitivity as a biomarker remains to be seen; particularly as it is likely single AE values from global or regional sources may be insufficient to identify an individual as developmentally atypical for their age. AE may have potential as stratification biomarkers in conjunction with psychometric and/or clinical data.

In addition to limitations inherent to the methods of studies included in the review, there are limitations to the review itself. Given the sparsity of effect size measures for AE and age (age*AE correlations or age-related mean group differences between EOR and ECR AE) it was not possible to produce a meaningful meta-analytic measure of age-related AE change. In addition, our inclusive search approach (including searches for terms relating to fractal measures to ensure sufficient HE data was sourced to provide adequate converted comparisons) resulted in significant heterogeneity. The suitability of including infant AEs where participant attention was captured using “toys” (Carter Leno et al., 2022) as a “resting” AE measure could be disputed. However, we perceive this to be a necessary means to engage young infant participants and minimise motion, and in the trajectories we have qualitatively illustrated, these AE estimates are consistent with a trend of decreasing AEs from infancy towards childhood.

Whilst there is some evidence supporting the notion that 1/*f* measures *are* relevant to understanding E:I shifts (Gao et al., 2017), two key aspects bear consideration. Firstly, AE methods must be used within the contexts of the limits of data they are fitted to, many errors of which can be avoided by inspecting PSDs before fitting models to ensure oscillations are not masking the onset of the spectral plateau. Accordingly, this avoids missing or overparameterizing peaks (including using cutoffs where oscillations are only partially visible, as these will bias AE estimates) which in turn affect AE estimates and avoids over- or underfitting models. These suggestions have been raised previously by Gerster et al. (2022) and are immensely relevant given they impact interpretations of age-specific states of E:I balance. As Figure 8 of Gerster et al. (2022) demonstrates, FOOOF and IRASA are indeed highly comparable, although discrepancies are seen when high amplitude low-frequency peaks arise (<10 Hz) or multiple peaks overlap*,* and the spectral plateau onset ends prematurely. However, many such concerns can be mitigated by ample spectral screening and appropriate choice of the frequency range, peak width and height, and number of peaks selected. Further validation of such choices could also include the use of sensitivity analyses. Secondly, the spatial scale (regional, network or global) at which E:I is governed by 1*/f* and to what extent this occurs is yet unknown, as is the correspondence between pharmacological modulation of E:I *in vivo* and changes in 1/*f* (AE). Whilst administration of GABA_A_R agonists has been shown to increase delta bandpower and depending on the drug, power across other frequencies (Yamamoto *et al.,* 1985; Christian *et al.,* 2015), controlled pharmacological modulation of known excitatory and inhibitory targets does not always yield a shift in AE in the anticipated direction (ketamine: (Waschke et al., 2021; Salvatore et al., 2024), picrotoxin, bicuculline: (Salvatore et al., 2024), whilst in other cases, it corresponds with the known mechanism of action of the pharmacological agent (pentobarbital: (Salvatore et al., 2024), diazepam: (Gonzalez-Burgos et al., 2023; Salvatore et al., 2024), MK801: (Gonzalez-Burgos et al., 2023). Drug action on AE magnitude also appears to vary based on sleep state (Salvatore et al., 2024), Drug action on AE magnitude also appears to vary based on sleep state (Salvatore et al., 2024), for both induced (Leroy et al., 2023) or natural sleep (Favaro et al., 2023). Furthermore, in attributing a potential 1/*f* to E:I relationship based on neural sources, we must consider the co-modulation of 1/*f* owing to other physiological sources, such a recently highlighted link between brain 1/*f* and respiratory rhythm (Kluger et al., 2023).

## 5 Summary

In summary, this review demonstrates that age-related AE changes in early development are complex. However, there are significant gaps in the data which currently prevent the robust establishment of age-related directions of change and reliable AE ranges, particularly in infancy and toddlerhood. We identify consistent AEs across methods and scales and confirm higher values of ECR than EOR, as well as developmental changes in AE maxima. Our review exclusively characterises the maturation of AEs in the resting state. Thus, specific task-related AE changes across the lifespan remain to be explored. As AE data is made available to the community, we can collectively extend the findings of this review to advance knowledge of how E:I shapes FC in early development. Moreover, characterising typical AE development provides a point of reference for exploring atypical development in which early life E:I balance is perturbed, where AEs may serve as a potential non-invasive biomarker.

## Funding

R.A.S. and D.M. are supported by the 10.13039/501100000265Medical Research Council (MRC) and 10.13039/100009360King’s College London (KCL) as members of the Doctoral Training Partnership [MR/N013700/1]. C.L.E. is supported by the Institute for Translational Neurodevelopment. H.D. was supported by ADR UK (Administrative Data Research UK), an Economic and Social Research Council (ESRC) investment (part of UK Research and Innovation) [Grant number: ES/W002647/1] D.B. received support from a Wellcome Trust Seed Award in Science [217316/Z/19/Z]. T.A. received support from the MRC Centre for Neurodevelopmental Disorders, KCL [MR/N026063/1], an 10.13039/501100000265MRC translation support award [MR/V036874/1] and an MRC Senior Clinical Fellowship [MR/Y009665/1]. The authors acknowledge infrastructure support from the National Institute Health Research (NIHR) Maudsley Biomedical Research Centre (BRC) at South London and Maudsley NHS Foundation Trust and King’s College London. The authors also acknowledge support in part from the Engineering and Physical Sciences Research Council (EPSRC) Centre for Medical Engineering at Kings College London [WT 203148/Z/16/Z], 10.13039/501100000265MRC strategic grant [MR/K006355/1], the Department of Health and Social Care through an NIHR Comprehensive Biomedical Research Centre Award (to King’s College Hospital NHS Foundation Trust). The views expressed are those of the author(s) and not necessarily those of the NIHR or the Department of Health and Social Care.

## Declaration of Competing Interest

The authors declare that they have no known competing financial interests or personal relationships that could have appeared to influence the work reported in this paper.

## Data Availability

The data utilised for analyses discussed within this manuscript are not all freely available. Where data is open-source, we have marked this in the appropriate in-text and supplementary tables. Where data is not available, we have marked the original source(s), with specific reference to any supporting figures.
