# Supplementary material for: Aperiodic and Hurst EEG exponents across early human brain development: A systematic review
Source: Dev Cogn Neurosci. 2024 Jun 6;68:101402. doi: 10.1016/j.dcn.2024.101402 (PMC11254951; doi:10.1016/j.dcn.2024.101402)
Supplement: Supplementary file 1 — Supplementary material [file mmc1.docx]

Supplementary Materials for “Aperiodic and Hurst EEG exponents across early human brain development: a systematic review”

Stanyard, R. A.^1,2^, Mason, D.^3^, Ellis, C.^2^, Dickson, H.^2^, Short, R.^2^, Batalle, D.^1,2^, Arichi, T.^1,4,5,6^

^1^Centre for the Developing Brain, School of Biomedical Engineering and Imaging Sciences, King's College London, United Kingdom

^2^Department of Forensic and Neurodevelopmental Sciences, Institute of Psychiatry, Psychology and Neuroscience, King’s College London, United Kingdom

^3^ Social, Genetic, and Developmental Psychiatry Centre, Institute of Psychiatry, Psychology and Neuroscience, King’s College London, United Kingdom

^4^ Centre for Neurodevelopmental Disorders, King's College London, United Kingdom.

^5^ Children's Neurosciences, Evelina London Children's Hospital, Guy's and St Thomas' NHS Foundation Trust, United Kingdom.

^6^Department of Bioengineering, Imperial College London, United Kingdom.

***Supplement I. Systematic Review Example Search Strategy***

**Scopus Search Strategy, March 2023:**TITLE-ABS-KEY ( aperiodic AND ( exponent* OR slope ) ) OR TITLE-ABS-KEY ( aperiodic W/2 ( exponent* OR slope ) ) OR TITLE-ABS-KEY ( hurst AND exponent ) OR TITLE-ABS-KEY ( hurst W/2 exponent ) OR TITLE-ABS-KEY ( ( detrend* AND fluctuation AND analysis ) OR fractal ) AND TITLE-ABS-KEY ( eeg OR electroencephal* ) AND TITLE-ABS-KEY ( birth OR newborn OR neonat* OR infan* OR toddler* OR child* OR teenage* OR adolescent* OR ( young AND adult ) OR development* OR ( early AND life ) OR maturation* )

## **Supplementary Material II. QuADS Risk of Bias Criteria**

| **QuADS Criteria 0 1 2 3** | | | | | |
| --- | --- | --- | --- | --- | --- |
| 1. **Theoretical or conceptual underpinning to the research** | No mention at all. | General reference to broad theories or concepts that frame the study. e.g. key concepts were identified in the introduction section. | | Identification of specific theories or concepts that frame the study and how these informed the work undertaken. e.g. key concepts were identified in the introduction section and applied to the study. | Explicit discussion of the theories or concepts that inform the study, with application of the theory or concept evident through the design, materials and outcomes explored.  e.g. key concepts were identified in the introduction section and the application apparent in each element of the study design. |
| **2. Statement of research aim/s** | No mention at all. | Reference to what the sought to achieve embedded within the report but no explicit aims statement. | | Aims statement made but may only appear in the abstract or be lacking detail. | Explicit and detailed statement of aim/s in the main body of report. |
| **3. Clear description of research setting and target population** | No mention at all. | General description of research area but not of the specific research environment e.g. ‘in primary care.’ | | Description of research setting is made but is lacking detail e.g. ‘in primary care practices in region [x]’. | Specific description of the research setting and target population of study e.g. ‘nurses and doctors from  GP practices in [x] part of [x] city in [x] country.’ |
| **4. The study design is appropriate to address the stated research aim/s** | No research aim/s stated or the design is entirely unsuitable e.g. a Y/N item survey for a study seeking to undertake exploratory work of lived experiences. . | | The study design can only address some aspects of the stated research aim/s e.g. use of focus groups to capture data regarding the frequency and experience of a disease. | The study design can address the stated research aim/s but there is a more suitable alternative that could have been used or used in addition  e.g. addition of a qualitative or quantitative component could strengthen the design. | The study design selected appears to be the most suitable approach to attempt to answer the stated research aim/s. |
| **5. Appropriate sampling to address the research aim/s** | No mention of the sampling approach. | | Evidence of consideration of the sample required e.g. the sample characteristics are described and appear appropriate to address the research aim/s. | Evidence of consideration of sample required to address the aim. e.g. the sample characteristics are described with reference to the aim/s. | Detailed evidence of consideration of the sample required to address the research aim/s. e.g. sample size calculation or discussion of an iterative sampling process with reference to the research aims or the case selected for study. |
| **6. Rationale for choice of data collection tool/s** | No mention of rationale for data collection tool used. | | Very limited explanation for choice of data collection tool/s. e.g. based on availability of tool. | Basic explanation of rationale for choice of data collection tool/s. e.g.  based on use in a prior similar study. | Detailed explanation of rationale for choice of data collection tool/s. e.g. relevance to the study aim/s, codesigned with the target population or assessments of tool quality. |
| **7. The format and content of data collection tool is appropriate to address the stated research aim/s** | No research aim/s stated and/or data collection tool not detailed. | | Structure and/or content of tool/s suitable to address some aspects of the research aim/s or to address the aim/s superficially e.g. single item response that is very general or an open-response item to capture content which requires probing. | Structure and/or content of tool/s allow for data to be gathered broadly addressing the stated aim/s but could benefit from refinement.  e.g. the framing of survey or interview questions are too broad or focused to one element of the research aim/s. | Structure and content of tool/s allow for detailed data to be gathered around all relevant issues required to address the stated research aim/s. |
| **8. Description of data collection procedure** | No mention of the data collection procedure. | | Basic and brief outline of data collection procedure e.g. ‘using a questionnaire distributed to staff’. | States each stage of data collection procedure but with limited detail or states some stages in detail but omits others e.g. the recruitment process is mentioned but lacks important details. | Detailed description of each stage of the data collection procedure, including when, where and how data was gathered such that the procedure could be replicated. |
| **9. Recruitment data provided** | No mention of recruitment data. | | Minimal and basic recruitment data e.g. number of people invited who agreed to take part. | Some recruitment data but not a complete account e.g. number of people who were invited and agreed. | Complete data allowing for full picture of recruitment outcomes e.g. number of people approached, recruited, and who completed with attrition data explained where relevant. |
| **10. Justification for analytic method selected** | No mention of the rationale for the analytic method chosen. | | Very limited justification for choice of analytic method selected. e.g. previous use by the research team. | Basic justification for choice of analytic method selected e.g. method used in prior similar research. | Detailed justification for choice of analytic method selected e.g. relevance to the study aim/s or comment around of the strengths of the method selected. |
| **11. The method of analysis was appropriate to answer the research aim/s** | No mention at all. | | Method of analysis can only address the research aim/s basically or broadly. | Method of analysis can address the research aim/s but there is a more suitable alternative that could have been used or used in addition to offer a stronger analysis. | Method of analysis selected is the most suitable approach to attempt answer the research aim/s in detail  e.g. for qualitative interpretative phenomenological analysis might be considered preferable for experiences vs. content analysis to elicit frequency of occurrence of events. |
| **12. Evidence that the research stakeholders have been considered in research design or conduct.** | No mention at all. | | Consideration of some the research stakeholders e.g. use of pilot study with target sample but no stakeholder involvement in planning stages of study design. | Evidence of stakeholder input informing the research. e.g. use of pilot study with feedback influencing the study design/conduct or reference to a project reference group established to guide the research. | Substantial consultation with stakeholders identifiable in planning of study design and in preliminary work e.g. consultation in the conceptualisation of the research, a project advisory group or evidence of stakeholder input informing the work. |
| **13. Strengths and limitations critically discussed** | No mention at all. | | Very limited mention of strengths and limitations with omissions of many key issues. e.g. one or two strengths/limitations mentioned with limited detail. | Discussion of some of the key strengths and weaknesses of the study but not complete. e.g. several strengths/limitations explored but with notable omissions or lack of depth of explanation. | Thorough discussion of strengths and limitations of all aspects of study including design, methods, data collection tools, sample & analytic approach. |

## **Supplementary Material III. Risk of bias assessment for included studies**


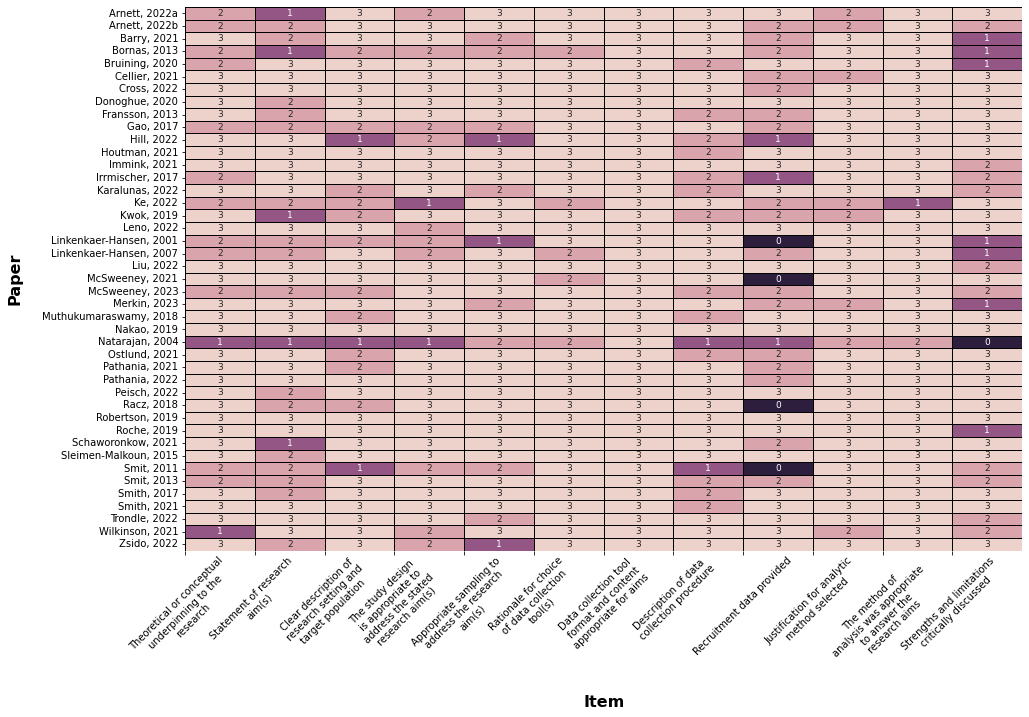

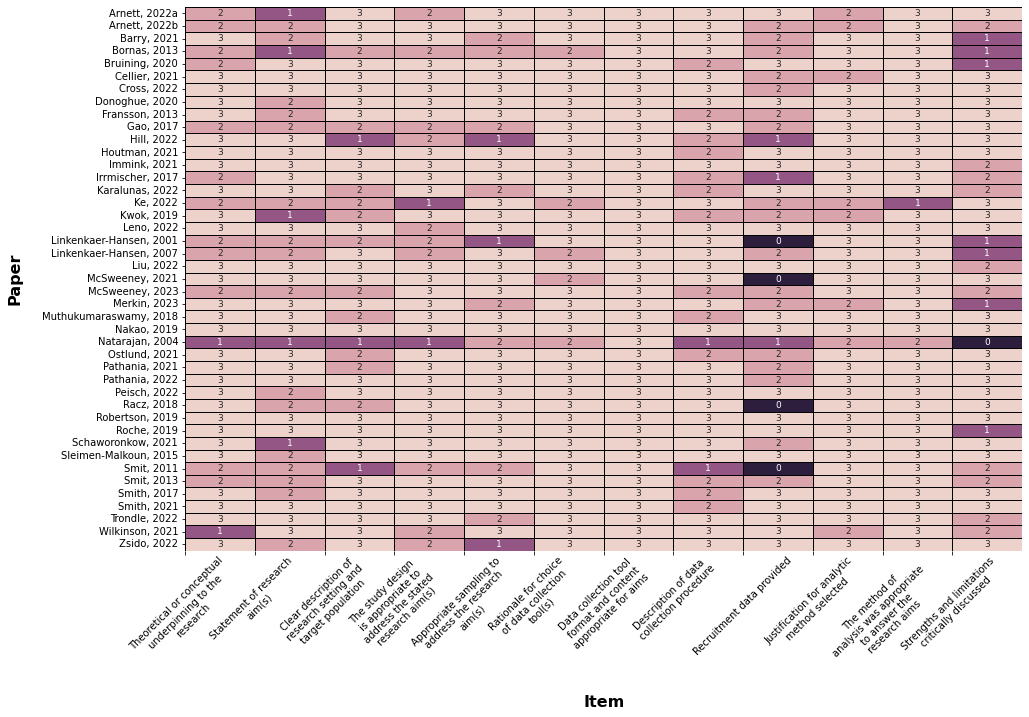


## **Supplementary Material IV. Studies included in the review, with technical details**

An extension of **Table 1**. (main text) with additional technical details regarding recording length, referencing, models and pertinent temporal windows for measure calculation (PSD estimation/sliding windows).

| **#** | **Study** | **Lifespan Stage (age, yrs)** | **Measure** | **Technical Specs** | **Scale(s)** | **Original Measure** | **HE to AE** | **Measure** | **N (M, F)** | **Source** | **F** |
| --- | --- | --- | --- | --- | --- | --- | --- | --- | --- | --- | --- |
| 1 | Schaworonkow & Voytek (2021) | Infancy  (0.10-0.56) | 1/f (FOOOF) | RECORDING, EPOCH  5 mins; 10s epoch length;  PSD ESTIMATION Multitaper REFERENCING Offline: Common average MODEL  1-10Hz; peak_width_limits=[0.5, 12.0], maximum_n_peaks=5, min_peak_height=0, peak_threshold=2.0, aperiodic_mode=‘fixed’. | Channelwise | S1: 1.74-3.22 (N = 20) S2: 1.74-2.95 (N = 20) S3: 1.79-2.25 (N = 20) S4: 1.94-2.98 (N = 5) S5: 1.46-2.76 (N = 3) S6: 1.88-2.63 (N = 2) |  | Baseline-wakeful reaching | 22(10,12) | Methods,  Auth Corr., GitHub |  |
| 2 | Karalunas (2022) | Infancy (0.12±0.01)  Adolescent (14.10±1.30) | 1/f (FOOOF) | RECORDING, EPOCH  8 mins; 2s epoch length  PSD ESTIMATION  Hanning window; no further information given  REFERENCING  Offline: Common average  MODEL  Infant 1-30Hz, Adolescent 2-50Hz  peak_width_limits=[1,8], maximum_n_peaks=6, min_peak_height=0, peak_threshold=2.0, aperiodic_mode=‘fixed’. | Global,  Channelwise | Infant EOR (PEACH cohort): 2.21±0.28 Adol EOR (1.85±0.28): ECR (1.98±0.26), EOR-ECR avg (1.91±0.28) Infant: 2.48±0.24 (Cz, EOR)  Adol: 2.28±0.19 (Cz, EOR),  2.33±0.27 (Pz, ECR), 2.29±0.19(EOR-ECR average) |  | EOR, ECR EOR-ECRavg | 69 (36,33) 152(85,67) | Auth Corr. |  |
| 3 | Fransson (2013) | Infancy (0.81, 0.75-0.85) | 1/f (PLE) | RECORDING, EPOCH Unknown; 5 mins PSD ESTIMATION, REFERENCING Unknown MODEL 0.2-30Hz | Global,  Regional, Channelwise | 2.07±0.22 |  | Natural Active/  Quiet Sleep | 15(9,12) | Fig 4 | Y |
| 4 | Carter-Leno (2022) | Infancy (0.90±0.05) | 1/f (FOOOF) | RECORDING, EPOCH 3 mins social, 3 mins non-social; 1s segment PSD ESTIMATION FFT; 1Hz bins REFERENCING Average  MODEL  1-10Hz. peak_width_limits=[2,8], maximum_n_peaks=4, peak_threshold=0.1, aperiodic_mode=‘fixed’. | Global, Regional, Channelwise | 1.50±0.13 (non-social), 1.52±0.16  Fz: social (1.53±0.16), non-social (1.51±0.13) Cz: social (1.51±0.15), non-social (1.49±0.13) Pz: social (1.51±0.16), non-social (1.49±0.13) |  | ~EOR (social and non-social videos) | 24(13,11) | Table 1,  Fig 4, Auth Corr. |  |
| 5 | Roche (2019) | Infancy (1.92-10.25) | 1/f (PLE) | RECORDING, EPOCH  5-10 mins; 1s, non-overlapping  PSD ESTIMATION  FFT, Hanning window.  REFERENCING  Online: vertex (Cz), Offline: Common average  MODEL  2-24Hz | Global^est^, Regional | ~0.58 |  | ~EOR (movie) | 37(0,37) | Methods, Results |  |
| 6 | Smith, R. (2021) | Infancy (med. 0.63, 0.43-0.82) | HE | RECORDING, EPOCH ~19h; > 2 wake, 2 sleep epochs; REFERENCING Linked ear HURST SCALES Box sizes 1- 1/10^th^ signal length (max 120s) | Global | Delta[1-3Hz]: ~0.80 (A), 0.68(S)  Theta[4-7Hz]: ~0.74(A), 0.68(S) Alpha[8-12Hz]: ~0.69(A), 0.68(S)  Beta[13-30Hz]: ~0.88(A), 0.72(S) |  | Awake, Sleep | 20(12,8) | Section 3.1,  Fig 6, Auth Corr. |  |
| 7 | Smith, R. (2017) | Infancy (med. 0.58, 0.48-0.94) | HE | RECORDING, EPOCH Unknown PSD ESTIMATION 50% window overlap REFERENCING  Unknown  HURST SCALES Log(3-25s) windows | Global ^s^ | Delta[1-3Hz]: ~0.78 Theta[4-7Hz]: ~0.70  Alpha[8-12Hz]: ~0.66  Beta[13-30Hz]: ~0.94 |  | Awake ~(EOR) | 21(?,?) | Fig 5, Auth Corr. |  |
| 8 | Cellier (2021) | Toddler (N=5),  Child (N=81), Adolescent (N=22), Young Adult (N=8) (2.95-24) | 1/f (FOOOF) | RECORDING, EPOCH Unknown length, 512ms PSD ESTIMATION Welch, 45% overlap, 512 and 1024ms sliding windows REFERENCING Average  MODEL  1-40Hz. peak_width_limits=[1, 2_MIPDB_ /4_SRS_], maximum_n_peaks=4, peak_threshold=2.0, aperiodic_mode=‘fixed’. | Regional (Parietal-midline [P], Frontal-midline [F]) | Toddler: [P] 1.45±0.23, [F] 1.32±0.54 Children: [P] 1.23±0.25, [F] 1.34±0.22 Adolescents: [P] 1.24±0.18, [F] 1.13±0.24 Young Adults: [P] 1.14±0.12, [F] 1.11±0.09 |  | EOR | 116 (33,24,59 unlabelled) | Fig 2,  Sections 2.2, 3.1,  Auth Corr., OSF |  |
| 9 | Houtman (2021) | Toddler (2.92 [N=8], 3.92 [N=13]),  Child (7-16[N=29]) | 1/f (FOOOF), HE | RECORDING, EPOCH  1-19 min length (multi-cohort); 1s epoch length;  PSD ESTIMATION Welch, 2s Hamming, 50% overlap REFERENCING mastoid reference (1/2 TD studies) MODEL  1-30Hz; peak_width_limits = [1,6], maximum_n_peaks = 6, min_peak_height = 0.05, peak_threshold = 1.5, aperiodic_mode = “fixed” HURST SCALES log(4)-log(20) (< 8Hz), log(2)-log(20) (>8Hz) | Global  Channelwise | Infant-toddler (I) & child-adol (C): HE, 11-18Hz: I: ~0.655, C: ~0.656 Infant-toddler (I) & child-adol (C): AE, ~1.11-1.60  (Hurst, 11-18Hz): I: ~0.63-.70), C: ~0.64 -0.74, 0.66±0.02 |  | EOR | 50 (28,22): Inf-Todd: 21 (14,7) Child-Adol: 29 (14,15) ^a^ | Fig 3, 5 Supp. Fig 5 |  |
| 10 | Wilkinson & Nelson (2021) | Child (3.98±1.09, 2.67-6.67) | 1/f (FOOOF) | RECORDING, EPOCH 2-5 mins; 2s segment PSD ESTIMATION Multi-tapering, 4s window, 1s step size REFERENCING Online: Cz; Offline: average. MODEL 2-55Hz. peak_width_limits=[1, 18], maximum_n_peaks=7, peak_threshold=2.0, aperiodic_mode=‘fixed’. | Global Regional | 1.19±0.12  Frontal: 1.26±0.13  Central: 1.33±0.14  Temporal: 1.11±0.15  Posterior: 1.07±0.32 |  | EOR | 12(12,0) | Methods,  Results, Auth Corr. |  |
| 11 | Robertson (2019) | Child (5.65±1.23) | 1/f (FOOOF) | RECORDING, EPOCH  1-2 min length; 1s epoch length;  PSD ESTIMATION Welch, 1s Hamming, 50% overlap REFERENCING Common average  MODEL  4-50Hz. peak_width_limits=[1, 8], maximum_n_peaks=8, peak_threshold=2.0, aperiodic_mode=‘fixed’. | Global Channelwise | 1.51±0.32 |  | EOR | 50(36,14) | Table 1,  Fig 2A, B |  |
| 12 | McSweeney (2023) | Child (6.92±2.21) | 1/f (FOOOF) | RECORDING, EPOCH  3 min length; 2s epoch length;  PSD ESTIMATION Welch, 2s Hamming, 50% overlap Single 1-49Hz spectrum per sub REFERENCING mastoid reference (1/2 TD studies) MODEL  3-40Hz. peak_width_limits=[1, 8], min_peak_height=0.05, peak_threshold=0.5, max_n_peaks=6 | Global | EOR: 1.53±0.31 ECR: 1.77±0.28 |  | EOR, ECR | 502(230,272) | Section 3.2,  Auth Corr. |  |
| 13 | Arnett (2022a) …Stein | Child (8.83±1.23) | 1/f (FOOOF) | RECORDING, EPOCH 85-120s; continuous segment PSD ESTIMATION Welch, 1s Hamming, 50% overlap REFERENCING online: vertex (Cz); offline: Common average  MODEL  1-50Hz. peak_width_limits=[2,12], min_peak_height=0.5, peak_threshold=2.0, max_n_peaks=8 | Global | 1.77±0.15 (Median: 1.76) |  | EOR | 29(19,10) ^b^ | Methods, Auth Corr. |  |
| 14 | Arnett (2022b) … Levin | Child (8.83±1.23) | 1/f (FOOOF) | RECORDING, EPOCH 85-120s; continuous segment  PSD ESTIMATION Welch, 1s Hamming, 50% overlap REFERENCING Online: vertex (Cz); Offline: Common average MODEL  1-50Hz. peak_width_limits=[2,12], min_peak_height=0.5, peak_threshold=2.0, max_n_peaks=8 | Global | 1.77±0.15 (Median: 1.76, range: 0.22-2.30) |  | EOR | 29(19,10) ^b^ | Methods, Auth Corr. |  |
| 15 | Peisch & Arnett (2022) | Child (9.40±1.36) | 1/f  (FOOOF) | RECORDING, EPOCH  85s length; continuous segment PSD ESTIMATION Welch, 1s Hamming, 50% overlap REFERENCING Online: vertex (Cz); Offline: Common average  MODEL  1-50Hz. peak_width_limits=[2,12], min_peak_height=0.5, peak_threshold=2.0, max_n_peaks=8 | Global Regional | 1.78±0.14  Anterior Frontal (AF): 1.79±0.14 Frontal (FR): 1.79±0.13 Central (CE): 1.75±0.15 Parietal (PR): 1.81±0.16 Occipital (OC): 1.77±0.22 |  | EOR | 29(19,10) ^b^ | Methods,  Auth Corr. |  |
| 16 | Hill (2022) | Child (9.41±1.95) | 1/f (FOOOF) | RECORDING, EPOCH 2 mins ECR, 2 mins EOR; 2s PSD ESTIMATION Hamming window 50% overlap, 2s REFERENCING Online: Cz, Offline: common average MODEL  1-40Hz. peak_width_limits=[1,12], min_peak_height=0.0, peak_threshold=2.0, max_n_peaks=8 | Global  Regional (anterior [A], central [C], posterior [P]) | EOR: 1.65±0.18 ECR: 1.81±0.16  EOR: A (1.64±0.19), C (1.69±0.19)  P (1.68±0.20)  ECR: A (1.81±0.17), C (1.85±0.16),  P (1.84±0.18) |  | EOR, ECR | 139 (72, 67) | Fig 2, Auth Corr. |  |
| 17 | Trondle (2022) | Child (N=153),  Adolescent (N=34),  Young Adult (N=3) (10.07±3.39, 5.02-21.67) | 1/f  (FOOOF) | RECORDING, EPOCH EO 20s; 1m 40s total, EC 40s; 3m 20s total; 2s epochs; averaged PSD ESTIMATION Welch, 2s sliding windows, 0.25Hz resolution REFERENCING Online: Cz, Offline: common average MODEL  peak_width_limits=[0.5,12], min_peak_height=0.0, peak_threshold=2.0, max_n_peaks=inf, aperiodic_mode=‘fixed’ | Regional (Parieto-occipital) | 1.89±0.36 (0.68-2.77) Child: 1.98±0.30  Adolescent: 1.58±0.37  Young adult: 1.12±0.04 |  | ECR | 190 (104,86) | Methods, Auth Corr., Fig 3, App. 4,  Supp. 2, 3 |  |
| 18 | Kwok (2019) | Child 4yrs (N=8),  5yrs (N=14),  6yrs (N=11), (5.60±?.??) | HE | RECORDING, EPOCH 3 min EO EC; 60 x 20s epochs REFERENCING Bilateral mastoid average HURST SCALES log(1-19.5s) | Global,  Channelwise ^est^ | Median: ~0.09 (EOR) ~0.06(ECR)  Posterior electrodes: ~0.09 EOR, ECR |  | EOR, ECR | 33(?,?) | Fig 6A-C |  |
| 19 | Smit (2011) | Child (5.27±0.19, 6.79±0.19), Adolescent (16.06±0.55 17.57±0.55), Young Adult (26.18±4.15) (5-50) | HE | RECORDING, EPOCH  3-4mins;  REFERENCING Earlobes HURST SCALES log windows(1.5-20s) | Channelwise (12 channels) | Child Theta (P3 maxima): 0.77±0.09 (5yrs), 0.76±0.07 (7yrs)  Child Alpha (O2 maxima): 0.70±0.09 (5yrs), 0.71±0.08 (7yrs) Child Beta: 0.64±0.09 (5 yrs, Fp2), 0.62±0.08 (7yrs, F8)  Adol Theta (Fp1 maxima): 0.72±0.06 (16yrs), 0.72±0.06 (18yrs) Adol Alpha (O1 maxima): 0.72±0.10 (16yrs), 0.73±0.12 (18yrs) Adol Beta: 0.64±0.09 (16yrs), 0.66±0.11 (18yrs)  YA Theta (F3 maxima): 0.73±0.07 (25yrs)  YA Alpha (P4 maxima): 0.75±0.09 (25yrs)  YA Beta (O1 maxima): 0.67±0.10 (25yrs) |  | ECR | 5yrs 366 7yrs 378 16yrs 426 18yrs 387 25yrs 396 | Auth Corr.  Methods, Fig 3, Table 2 |  |
| 20 | Bruining (2020) | Child (10.30±1.54) | HE | RECORDING, EPOCH 3-5 mins ECR;  REFERENCING Online: Common mode sense, Offline: common average HURST SCALES 2-30s | Global | 0.66±0.04 |  | ECR | 29 (14,15)^a^ | Supp. Table 1 | Y |
| 21 | McSweeney (2021) | Adolescent (12-17) | 1/f (FOOOF) | RECORDING, EPOCH 3 mins ECR, 3 mins EOR; 3s PSD ESTIMATION Hanning taper, resolution 0.33Hz REFERENCING Online: Cz, Offline: Common average MODEL 1-45Hz. peak_width_limits=[1,6], min_peak_height=0.0, peak_threshold=2.0, max_n_peaks=4, aperiodic_mode=‘fixed’ | Global (1-45Hz) | t_1 (all subjects)_: EOR (1.21±0.30)  t_1 (subjects with t1 & t2)_: EOR (1.21±0.30)  t_1 (all subjects)_: ECR (1.33±0.27)  t_1 (subjects with t1 & t2)_: ECR (1.21±0.30)  t_2 (all subjects)_: EOR (1.10±0.26)  t_2 (subjects with t1 & t2)_: EOR (1.11±0.27)  t_2 (all subjects)_: ECR (1.16±0.25)  t_2 (subjects with t1 & t2)_: ECR (1.17±0.26) |  | EOR, ECR | 186 (85,101)  95 @t_1_, t_2_ | Fig 1B,  Results |  |
| 22 | Ostlund (2021) | Adolescent (13.97±1.28) | 1/f (FOOOF) | RECORD, EPOCH 4mins EO, 4 mins EC; unknown  PSD ESTIMATION  Fourier transform, 0.5Hz increm. 1-50Hz  REFERENCING  Online: Cz, Offline: average. MODEL 2-50Hz*.* peak_width_limits=[1,8], max_n_peaks=8, aperiodic_mode=‘fixed’ | Global (2-50Hz) | (EOR+ECR/2): 1.80±0.28 (0.92-2.57)  EOR: 1.72±0.31 (0.88-2.49)  ECR: 1.88±0.28 (0.90-2.65) |  | EOR, ECR | 97(53,43) | Table 1 |  |
| 23 | Linkenkaer-Hansen (2007) | Adolescent (16.50-19.50) | HE | RECORDING, EPOCH  3-6mins; NA  REFERENCING  Online: Linked earlobes, Offline: Common average  HURST SCALES 1-20s | Channelwise (Alpha, Beta) | Alpha: (0.70-0.74±0.08-0.11) Beta:(0.61-0.66±0.07-0.09) |  | ECR | 390 (196,194) | Table 1 |  |
| 24 | Gao, F. (2017) | Adolescent (18.30±2.80) | HE | RECORDING, EPOCH  5 mins; 2 mins  REFERENCING  Online: Mastoid average  HURST SCALES log(1-15s) | Channelwise (Delta-Gamma)^est^ | Alpha: ~0.80 Beta: ~0.70 | 0.60 0.40 | ECR | 15(15,0) | Fig 2 |  |
| 25 | Donoghue (2020) | Young Adult (19.56±1.90) | 1/f (FOOOF) | RECORDING, EPOCH  2 min  PSD ESTIMATION  Welch, 2s windows, 50% overlap  REFERENCING  Offline: Common average  MODEL  2-40Hz. peak_width_limits =[1,6], max_n_peaks =6, min_peak_height =0.05, peak_threshold =1.5, aperiodic_mode =‘fixed’ | Channelwise (Cz) | 1.43±0.25 |  | EOR | 16(8,8) | Auth Corr., Results |  |
| 26 | Linkenkaer-Hansen (2001) | Young Adult (20-30) | 1/f (PLE)  HE | RECORDING, EPOCH 20 mins; 120s window PSD ESTIMATION 0.005-0.5 Hz  REFERENCING  Unknown HURST WINDOW  Unknown | Global (Alpha [8-13Hz]) 4-channel avg | PLE ECR: 0.36±0.17  PLE EOR: 0.51±0.12  HE ECR: 0.68±0.07  HE EOR: 0.70±0.04 |  | EOR, ECR | 10(9,1) | Results | Y |
| 27 | Muthukumaraswamy & Liley (2018) | Young Adult (23.00±??) | 1/f (IRASA) | RECORDING, EPOCH 5 min; 10s segments PSD ESTIMATION Hanning window, FFT length “next a^2^ after multiplying sub-epoch data length * max(*h*)” REFERENCING Online: Common average, Offline: FCz. MODEL resampling factor *h* 1.1-2.9 (0.05 steps); β_lf_ 0.1-2.5Hz β_hf_ 20-100Hz | Global,  Channelwise | β_lf_ 1.36(1.12-1.72) β_hf_ 1.48(1.18-1.81) β_lf_  frontal maxima: 1.72 β_hf_  central maxima: 1.81 |  | ECR | 17(17,0) | Methods, Supp. Fig 7 |  |
| 28 | Pathania (2021) | Young Adult (20.88±2.24) | 1/f (PLE)  1/f (FOOOF) | RECORDING, EPOCH  2 mins; 1s segments  PSD ESTIMATION  Welch, 50% overlap; FFT 0.977Hz bins, Hamming 50% taper.  REFERENCING  Online: Left earlobe, Offline: Ear average  MODEL  2-25Hz. | Global (FOOOF), Regional (FOOOF), | 1.36±0.26 F(1.18±0.34), C(1.40±0.28),  P(1.46±0.28), O(1.41±0.29) |  | EOR | 59(19,40) | Auth Corr. |  |
| 29 | Barry (2021) | Young Adult (21.20±3.80) | 1/f PN Slope (PaWNextra) | RECORDING, EPOCH  4min EOR; 2min ECR; 2s segments  PSD ESTIMATION  Hanning window 10% overlap, DFT correction factor 1.0529  REFERENCING  A1  MODEL  0.5Hz extrapolated to 2-24Hz, Ln(Power) 1Hz | Global  Channelwise (30 channels) | EOR (session 1, 2 average): 1.07±0.33 ECR: 1.22±0.38 EOR: 0.41-1.50 (Fp1, Cz) ECR: 0.38-1.22 (Fp1, C4) |  | EOR, ECR | 20(3,17) | Auth Corr. |  |
| 30 | Merkin (2023) | Young Adult (22.20±3.90,18–35) | 1/f (FOOOF) | RECORDING, EPOCH  2 mins; 2s  PSD ESTIMATION  Welch, 2s Hamming window, 50% overlap  REFERENCING  Online: Common average  MODEL  2–40 Hz. aperiodic_mode=‘fixed’ mode. peak_width_limits = [1,12], maximum_n_peaks = 6, peak_threshold = 2, min_peak_ height = 0 | Global ^est^ Regional | ~1-2.1 ~ range 1.3-1.6 YA |  | ECR | 85(37,48) | Sections 2.1, 3.1, Supp. S5 |  |
| 31 | Ke (2022) | Young Adult (22.29±2.28) | 1/f (FOOOF) | RECORDING, EPOCH  Unknown; 2mins  PSD ESTIMATION  FFT 0.5-30Hz.  REFERENCING  Online: FCz, Offline: Mastoid average  MODEL  peak_width_limits = [1.0,5.0], max_n_peaks = 6, min_peak_height = 0.1, peak_threshold = 1, aperiodic_mode = 'fixed' | Global,  Regional (Frontal, Central, Parietal, Occipital) | Global (1.84±0.34) Frontal (1.99±0.35) Central (1.84±0.34) Parietal (1.76±0.37) Occipital (1.67±0.52) |  | EOR | 90(44,46) | Table 1,  Auth Corr. |  |
| 32 | Smit (2013) | Young Adult (22.40, 21–25) | 1/f (PLE) HE | RECORDING, EPOCH Unknown; 6 mins PSD ESTIMATION Welch, 75% overlap, Hanning envelope 64s. REFERENCING  Common average MODEL  0.0156-2Hz HURST WINDOW  0.5-64s (0.0026-.067Hz) | Channelwise (Alpha [9-13Hz])  CP3 | Maxima (both): central midline, scalp ranges Hurst (0.70-0.80), 1/f (0.20-0.40)  PLE = 0.43 HE = 0.66 (Range: 0.66-1.04) |  | EOR | 39(11,28) | Fig 1B/C,  Auth Corr. | Y |
| 33 | Zsido (2022) | Young Adult (22.48±3.79) | 1/f (FOOOF) | RECORDING, EPOCH 11min; continuous recording PSD ESTIMATION  Welch, 4s window, 50% overlap REFERENCING  Online: Right mastoid, Offline: Common average  MODEL  1-40Hz. FOOOF parameters unspecified. | Global ^est^ | ~1.40 |  | ECR | 31(?,?) | Methods |  |
| 34 | Immink (2021) | Young Adult (22.67±3.85) | 1/f (IRASA) | RECORDING, EPOCH  5 mins ECR, 5 mins EOR; 30s ECR  PSD ESTIMATION  IRASA  REFERENCING  Online: FCz, Offline: Mastoid average  MODEL  0.1-40Hz, resampling factor 1.1-1.9 (0.05 steps) | Global | 2.06±0.13 (range: 1.82-2.48) |  | ECR | 45(22,23) | Section 3.1,  Auth. Corr. |  |
| 35 | Pathania (2022) | Young Adult (23.29±3.47) | 1/f (FOOOF) | RECORDING, EPOCH  2 mins; 1s segments  PSD ESTIMATION  Welch, 50% overlap; FFT 0.977Hz bins, Hamming 50% taper.  REFERENCING  Online: right ear.  MODEL  2-25Hz. peak_width_limits**=**[1.0, 8.0], max_n_peaks**=**8, min_peak_height**=**0.05, peak_threshold**=**2.0, aperiodic_mode**=**'fixed' | Global (2-25Hz), Regional | 1.17±0.23 F(1.20±0.25), C(1.22±0.27), P(1.09±0.28), O(0.96±0.28) |  | ECR | 21(11,10) | Section 4.1,  Figure 2B,  Auth Corr. |  |
| 36 | Cross (2022) | Young Adult (25.00±7.13) | 1/f (FOOOF, IRASA) | RECORDING, EPOCH  2 mins ECR, EOR; 13.2s segments  PSD ESTIMATION  (FOOOF-only) Welch, Hann-tapered, zero-padded to 2048 points  REFERENCING  Online: FCz, Offline: mastoid average  MODEL *IRASA*  *h =* 1.1-1.95 (0.05 steps) *FOOOF* 1-35Hz. peak_width_limits=[1, 12], maximum_n_peaks=infinite, min_peak_height=0, peak_threshold=2.0, aperiodic_mode= ‘fixed’ | Global | *IRASA* ECR: 1.11±0.30 *IRASA* EOR: 1.08±0.31 |  | EOR, ECR | 35 (18,17) | Auth Corr. |  |
| 37 | Nakao (2019) | Young Adult (19.57±?? 18-21) | HE | RECORDING, EPOCH 5 mins; 30s segments PSD ESTIMATION 75% overlap REFERENCING Offline: Common average HURST WINDOW  log(1-30s) | Alpha [8-13Hz] | FCz: 0.75±0.12  Min (T7, 0.74±0.12) Max (O1, 0.80±0.13) | 0.50  (0.48-0.60) | ECR | 23(11,12) | Fig 5,  Section 3.3,  Auth Corr. |  |
| 38 | Natarajan (2004) | Young Adult (20.00±3.00) | HE | RECORDING, EPOCH 20 mins; 5 mins REFERENCING  Unknown HURST SCALES  Unknown | Global (1-50Hz) | 0.29±0.06 | -0.42 | ECR | 30(15,15) | Table 1 |  |
| 39 | Liu, S (2022) | Young Adult (20-30) | HE | RECORDING, EPOCH 4 mins ECR, 4 mins EOR; 5s segments PSD ESTIMATION Welch, Hamming window, 50% overlap REFERENCING  Online: Linked left mastoid (M1), Offline: Average mastoid HURST WINDOW  Unknown | Channelwise ^est^ (Broadband) [0.5-120Hz] | EOR ~0.80-0.82 | EOR 0.60-0.64 | ECR, EOR | 26(?,?) | Fig 5 |  |
| 40 | Sleimen-Malkoun (2015) | Young Adult (22.70±1.60, 18.80-25.10) | HE | RECORDING, EPOCH 1.5 min ECR, EOR; 10s segments  PSD estimation  2500pt Hanning window; 4096pt (zero-padded) REFERENCING Online: left mastoid, Offline: average mastoid HURST WINDOW  4-50ms, 16-200ms, 4ms steps | Global (0.5-100Hz) | 1.69 Higher for posterior vs midline | 2.38 | ECR | 31(17,14) | Fig 4 | Y |
| 41 | Irrmischer (2018) | Young Adult (25.00±6.20) | HE | RECORDING, EPOCH 5mins ECR, 5mins EOR REFERENCING Online: Cz, Online: Common average  HURST WINDOW  5-30s (delta, theta) 2-30s (alpha)  1-30s(beta)  1-30s (gamma) | Global (Delta [1-4Hz], Theta [4-8Hz], Alpha [8-13Hz], Beta [13-45Hz]) | ECR (N = 57) Theta: 0.66±0.01  Alpha: 0.71±0.01  Beta: 0.66 ± 0.01  EOR (N = 23)  Theta: 0.69±0.02  Alpha: 0.75±0.02  Beta: 0.70 ± 0.01 | ECR 0.32 0.42 0.32 EOR 0.38 0.50 0.40 | EOR, ECR | 57(22,35) | Results |  |
| 42 | Bornas (2013) | Young Adult (24.61±7.03) | HE | RECORDING, EPOCH 8 mins; 1 min segments (EOR, ECR) REFERENCING  Linked earlobes HURST WINDOW  0.1-0.6s (broadband),  1-6s (narrowband) | Regional (theta [3-7Hz], alpha [8-13Hz], broadband [1-40Hz]): Central [C], Parietal [P], Occipital [O]) | Theta  C(0.75±0.07), P(0.76±0.07), O(0.74±0.07)  Alpha C(0.76±0.07), P(0.80±0.08), O(0.85±0.10)  Broadband C(0.85±0.07), P(0.86±0.06), O(0.88±0.06) | Theta 0.50,0.52 0.48 Alpha 0.52, 0.60 0.70 Broadband 0.70, 0.72 0.76 | EOR, ECR average | 56(20,36) | Table 1 |  |

## **Supplementary Material V.** Regional AE follows similar trends to Global AE.

## (A) Regional AE trends are similar to those observed with global data in **Figure 2A**. However ROI AE data across methods are sparse. Towards young adulthood (YA) converted HE border the lower bound of FOOOF AE estimates. PLE estimates differ in magnitude for reasons discussed elsewhere. (B) Overlay of global and regional AE data across the studied period. Colours correspond to lifespan stage, as in **Figure 3B**.
